# House ammonia exposure causes alterations in microbiota, transcriptome, and metabolome of rabbits

**DOI:** 10.3389/fmicb.2023.1125195

**Published:** 2023-05-12

**Authors:** Keyao Li, Shuo Pang, Zhechen Li, Xiaoning Ding, Yating Gan, Qianfu Gan, Shaoming Fang

**Affiliations:** College of Animal Science (College of Bee Science), Fujian Agriculture and Forestry University, Fuzhou, China

**Keywords:** house ammonia, microbiota, transcriptome, metabolome, rabbit

## Abstract

**Introduction:**

Pollutant gas emissions in the current production system of the livestock industry have negative influences on environment as well as the health of farm staffs and animals. Although ammonia (NH3) is considered as the primary and harmful gas pollutant in the rabbit farm, less investigation has performed to determine the toxic effects of house ammonia exposure on rabbit in the commercial confined barn.

**Methods:**

In this study, we performed multi-omics analysis on rabbits exposed to high and low concentration of house ammonia under similar environmental conditions to unravel the alterations in nasal and colonic microbiota, pulmonary and colonic gene expression, and muscular metabolic profile.

**Results and discussion:**

The results showed that house ammonia exposure notably affected microbial structure, composition, and functional capacity in both nasal and colon, which may impact on local immune responses and inflammatory processes. Transcriptome analysis indicated that genes related to cell death (*MCL1, TMBIM6, HSPB1*, and *CD74*) and immune response (*CDC42, LAMTOR5, VAMP8*, and *CTSB*) were differentially expressed in the lung, and colonic genes associated with redox state (*CAT, SELENBP1, GLUD1*, and *ALDH1A1*) were significantly up-regulated. Several key differentially abundant metabolites such as L-glutamic acid, L-glutamine, L-ornithine, oxoglutaric acid, and isocitric acid were identified in muscle metabolome, which could denote house ammonia exposure perturbed amino acids, nucleotides, and energy metabolism. In addition, the widespread and strong inter-system interplay were uncovered in the integrative correlation network, and central features were confirmed by in vitro experiments. Our findings disclose the comprehensive evidence for the deleterious effects of house ammonia exposure on rabbit and provide valuable information for understanding the underlying impairment mechanisms.

## Introduction

The current production system of the livestock industry is characterized by increasing numbers of animals raised in largely confined housing facilities (Alvarado and Predicala, 2019). Although this improves the industry's productivity and profitability, various environmental issues have been raised. Pollutant gas emissions from animal barns not only affect the surrounding environment but also threaten the health of farmers and the wellbeing of animals (Kilic et al., 2021). Among the main gases emitted from the livestock production industry, ammonia is produced during manure management, and its concentration is typically high in confined livestock houses with bad ventilation.

Microbiota colonized in the respiratory and intestinal tracts of farm animals establish a symbiotic relationship with the host and exert crucial modulatory roles in immune processes, neural functions, and endocrine pathways, which have important implications for the health and welfare of farm animals (Chen S. et al., 2021). Numerous studies have been conducted on farm animals raised in respiration chambers to evaluate the microbial and physiological alterations induced by ammonia exposure. For instance, sustained exposure to ambient ammonia causes pulmonary microbial perturbation in chickens, which promotes the release of inflammatory cytokines and suppresses the production of neurotransmitters via the lung–brain axis (Wang et al., 2022). Ammonia exposure disrupts chicken gut microbial homeostasis, activating the TLR4/TNF-α signaling pathway and inducing intestinal inflammation (Zhou et al., 2021). Ammonia stress resulted in the increased presence of harmful bacteria in the nasal microbiota and decreased respiratory immunity, which is harmful to porcine growth performance (Wang et al., 2019). Inhalation of excessive ammonia contributes to the dysbiosis of the porcine gut-brain axis by interfering with the oxidative stress-inflammation-apoptosis interaction network (Li et al., 2021a). Although rabbits are particularly sensitive to ambient ammonia, to date, research focusing on the deleterious effects of ammonia on rabbits is limited (Cui et al., 2021). More importantly, the artificially ammoniated environments in the respiration chambers may fail to reflect the complicated air conditions of confined barns (Pokharel et al., 2017).

Thus, we selected rabbits raised in a commercial, confined barn as research subjects exposed to high- and low-level house ammonia under similar environmental conditions. Alterations in the microbiota, transcriptome, and metabolome of different tissues were investigated using multi-omics analysis. Our observations could provide valuable insights into the adverse role of house ammonia exposure in rabbits and lay the foundation for illustrating the underlying toxicity mechanism of house ammonia exposure.

## Materials and methods

### Experimental animals and environmental parameter measurements

A total of 648 weaned Ira rabbits (330 males and 318 females) were randomly assigned to 324 double-deck cages (two rabbits per cage) in a commercial, confined rabbit house of Laidewang Animal Husbandry Co., Ltd., Sanming, China. The rabbit house building is 57.7 m long, 6.6 m wide, and 3.8 m high and is equipped with mechanical ventilation and a cooling system (Supplementary Figure S1). During a 45-day experimental period in the summertime, a multi-gas analyzer (MultiRAE IR Lite, RAE Systems, USA) with electrochemical and NDIR sensors was used for NH_3_, H_2_S, and CO_2_ concentration measurements. The device recorded concentrations for 12 h (6:00 a.m.−6:00 p.m.) on a daily basis. Indoor air temperature, relative humidity, and air velocity in the house were measured at 3-h intervals throughout the experiment using a portable air velocity meter (PCE-VA 20, PCE, Spain). At the end of the experiment, each of the six rabbits (three male and three female) exposed to high and low levels (16.49 ± 1.67 vs. 4.81 ± 0.92 ppm) of house ammonia, but with similar other environmental parameters, were selected for sampling (Supplementary Table S1). A commercial pellet diet (Supplementary Table S2) was provided to all rabbits two times a day. All rabbits were healthy and did not receive any antibiotic, anticoccidial drug, probiotic, or prebiotic treatment before sampling.

### Sample collection

Nasal swabs were collected from the nares of the selected rabbits and placed into sterile tubes. The swabs were transported to the laboratory, where they were resuspended in 300 μl PBS and stored at −20°C for further analysis. The rabbits were anesthetized using electric shock and euthanized using exsanguination from the carotid artery. The lung, longissimus dorsi muscle, colon, and colonic digesta were immediately sampled after euthanization and frozen in liquid nitrogen for transportation. In the laboratory, the tissue and digested samples were stored at −80°C for further analysis. All animal experiments were conducted according to the guidelines for the care and use of experimental animals established by the Ministry of Agriculture and Rural Affairs of China. The project was specially approved by the Animal Care and Use Committee (ACUC) at Fujian Agriculture and Forestry University (NO. PZCASFAFU22020).

### 16S rRNA gene sequencing

According to the manufacturer's instructions, total microbial genomic DNA was extracted from nasal swabs and colonic digesta using a FastDNA SPIN Kit for Soil (MP Biomedicals, USA). DNA quantity and quality were detected using a Nanodrop ND-2000 spectrophotometer (Thermo Fisher Scientific, USA) and 1.5% agarose gel electrophoresis, respectively. The V3-V4 hypervariable regions of the 16S rRNA gene were amplified using the barcoded fusion primers 341F (5′-CCTACGGGNGGCWGCAG-3′) and 806R (5′- GGACTACHVGGGTATCTAAT-3′). The protocols for 16S rRNA gene sequencing and bioinformatics analysis were described in our previous research (Fang et al., 2020).

### Transcriptome sequencing

The total RNA in lung and colon samples was isolated using TRIzol reagent (Invitrogen, USA). RNA concentration, purity, and integrity were measured using the Nanodrop ND-2000 spectrophotometer (Thermo Fisher Scientific, USA) and a Bioanalyzer 2100 system (Agilent Technologies, USA). Following the manufacturer's illustrations, the sequencing libraries were constructed using a NEBNext^®^ UltraTM RNA Library Prep Kit for Illumina^®^ (NEB, Beverly, MA, USA). The prepared libraries were sequenced on an Illumina HiSeq 2500 platform using a 150-bp paired-end read strategy. Raw data generated from Illumina sequencing were subjected to quality control using FASTX-Toolkit software. After removing adaptor sequences and low-quality reads, the remaining clean data were aligned with the rabbit reference genome (*OryCun*2.0.107) using TopHat v2.0.14 software. HTSeq v0.6.0 was used to count the number of mapped reads to each gene, and the gene expression was calculated in fragments per kilobase of exon per million mapped fragments (FPKM) using Cufflinks v0.14.0 software. DESeq2 v1.16.1 software was used to identify the differentially expressed genes (DEGs) according to the thresholds of |log2(fold change)| > 1 and FDR-adjusted *P*-value of < 0.01. Gene ontology (GO) and Kyoto Encyclopedia of Genes and Genomes (KEGG) pathway enrichment analysis of DEGs was performed using the clusterProfiler R package.

### Muscle metabolomics profiling

Hundred milligram of muscle sample was homogenized in 1 ml of precooled extraction mixture (methanol: water, 1:1) using a high-throughput tissue grinder and ultrasonicated at room temperature for 25 min. After centrifugation at 13,000 rpm for 20 min at 4°C, 500 μL of supernatant was dried in a vacuum, resolved in 200 μl of 15% methanol, and filtered through a 0.2 μm membrane (Millipore, USA). The filtrate was collected for subsequent UPLC-QTOFMS analysis, and an aliquot of equal volume (20 μl) for each sample was mixed to make a quality control (QC) sample. An Acquity UPLC system (Waters, USA) equipped with an HSS T3 column (150 × 2.1 mm, 1.8 μm) was used for chromatographic separation. After separation by UPLC, a Q-TOF Premier (Waters, USA) equipped with an electrospray ionization (ESI) source operating in positive and negative modes with spray voltages of 3.5 and −2.5 kV was used for mass spectrometry analysis. System control and data acquisition were performed using MassLynx (Waters, USA). The raw data were processed using Progenesis QI (Waters, USA) for peak alignment to obtain a peak list consisting of the retention time, m/z, and peak area. The peaks were normalized to the QC sample using the MetNormalizer R package. The metabolites were identified by aligning against the HMDB database. Principal component analysis (PCA) and partial least squares discriminate analysis (OPLS-DA) were performed using the ropls R package. Differentially expressed metabolites were identified by variable importance in the projection (VIP) > 1 and by an FDR-adjusted *P*-value of < 0.05. A KEGG pathway enrichment analysis of differentially expressed metabolites was performed using the MetaboAnalyst 4.0 web server.

### Validation of key features

To confirm the key differentially enriched microbes and differentially expressed genes, quantitative PCR analysis was performed using microbial genomic DNA and tissue RNA, respectively. The PCR reactions were run in a 7500-Fast Real-Time PCR System (ABI, USA) using an SYBR^®^ Premix Ex Taq™ II Kit (TaKaRa, Japan). The PCR conditions comprised an initial denaturation at 95°C for 10 s, followed by 40 cycles of denaturation at 95°C for 5 s and annealing at 60°C for 30 s. The expression levels of microbes and genes were determined based on normalization to the 16S rRNA gene and β-actin using the 2^−Δ*ΔCt*^ method. The primer sequences are listed in Supplementary Table S3. The centrally differentially abundant metabolites were verified using targeted quantitative metabolomics methodology on a GC-MS/MS platform (Agilent Technologies, USA), as previously described (Shin et al., 2019). In brief, proteins were precipitated using acetonitrile from each 100 mg muscle sample containing 3,4-dimethoxybenzoic acid (0.1 μg) as an internal standard for isocitric acid and norvaline (0.2 μg) as an internal standard for L-glutamic acid. After centrifugation, the supernatant was adjusted to a pH ≥ 12 with 5.0 M sodium hydroxide in dichloromethane (1.5 ml) containing ethyl chloroformate (30 μl), which was converted to the ethoxycarbonyl derivative and subsequently the methoxime derivative by reaction with methoxyamine hydrochloride at 60°C for 1 h. After washing with diethyl ether two times, the aqueous phase was acidified with 10% sulfuric acid (pH < 2.0), saturated with sodium chloride, and sequentially extracted with diethyl ether (3 mL) and ethyl acetate (2 mL). The extracts were evaporated to dryness by nitrogen. Prior to GC-MS/MS analysis, dry residues containing isocitric acid and L-glutamic acid were reacted with TEA (5 μl) and toluene (10 μl) at 60°C for 30 min to produce TBDMS derivatives. The derivatives were transferred to GC–MS/MS and quantified by multiple reaction monitoring (MRM) modes.

### Statistical analysis

A Wilcoxon rank sum test with false discovery rate (FDR) correction was performed to detect differences in microbial diversity indices, relative abundances of microbes at the phylum and genus levels, and functional capacities. Nasal microbial principal coordinate analysis (PCoA) and colonic microbial hierarchical clustering analysis of the unweighted pair-group method with arithmetic means (UPGMA) were performed to reveal the structural variation using the ape and facto extra R packages, respectively. The nasal and colonic differentially enriched OTUs were visualized using linear discriminant analysis effect size (LEfSe) analysis and Interactive Tree of Life (iTOL), respectively. The nasal and colonic differentially enriched functional items were exhibited using ComplexHeatmap and the ggplot2 R package, respectively. A Spearman correlation network was first generated based on potential biomarkers identified in each omic dataset of high-level house ammonia-challenged rabbits, and the core community was defined by the Girvan-Newman algorithm (Lv et al., 2019). Subsequently, the features of the core community in each network were selected for constructing the integrated network, and key features were identified according to the maximal clique centrality (Ke et al., 2019).

## Results

### House ammonia affects the nasal microbial communities of rabbits

The microbial diversity of the nasal microbiota was first analyzed. Compared to low-level house ammonia-exposed rabbits, both the Shannon and ACE index values of rabbits exposed to a high concentration of house ammonia significantly declined (Supplementary Figures S2A, B). The PCoA analysis result based on unweighted and weighted UniFrac distances showed that the individuals were distinctively separated (Supplementary Figures S2C, D).

**Figure 1:**
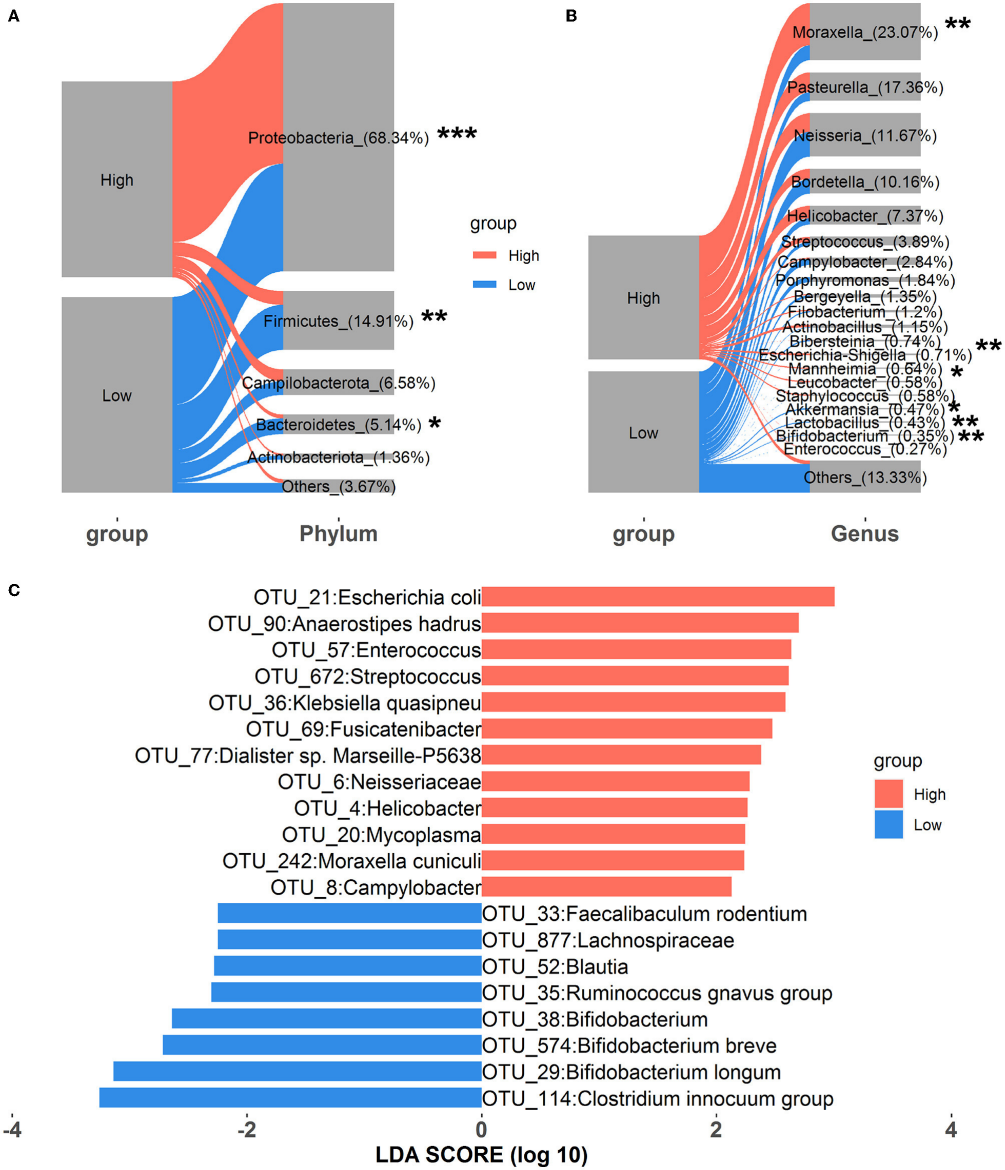
Alterations in the relative abundances of nasal microbial phyla **(A)**, genera **(B)**, and OTUs **(C)**. “*”, FDR-adjusted *P* < 0.05, “**”, FDR-adjusted *P* < 0.01, “***”, FDR-adjusted *P* < 0.005.

Alterations in nasal microbial composition were investigated at the phylum, genus, and OTU levels. As shown in Figure 1A, *Proteobacteria, Firmicutes, Campilobacterota, Bacteroidetes*, and *Actinobacteriota* were the top five phyla in all samples, and they constituted over 95% of the total sequences. Among these, the relative abundance of *Proteobacteria* significantly increased in rabbits exposed to high concentrations of house ammonia, and significant reductions in the relative abundances of both *Firmicutes* and *Bacteroidetes* were also observed. At the genus level, 20 dominant genera, including *Moraxella, Pasteurella, Neisseria, Bordetella, Helicobacter, Staphylococcus, Akkermansia, Lactobacillus, Bifidobacterium*, and *Enterococcus*, comprised more than 85% of the total sequences (Figure 1B). Of these, *Moraxella, Escherichia-Shigella*, and *Mannheimia* were more abundant in rabbits exposed to high concentrations of house ammonia, while *Akkermansia, Lactobacillus*, and *Bifidobacterium* exhibited lower abundances. As shown in Figure 1C, 12 OTUs were enriched in a high concentration of house ammonia-exposed rabbits, including one OTU annotated to the family *Neisseriaceae*, one OTU annotated to each of the genera *Enterococcus, Streptococcus, Fusicatenibacter, Helicobacter*, and *Mycoplasma*, and one OTU annotated to each of the species *Escherichia coli, Anaerostipes hadrus, Klebsiella quasipneu, Dialister sp. Marseille-P5638*, and *Moraxella cuniculi*. The other eight OTUs were augmented in low concentrations of house ammonia-exposed rabbits, including one OTU annotated to the family *Lachnospiraceae*, one OTU annotated to each of the genera *Clostridium innocuum* group, *Ruminococcus gnavus* group, *Bifidobacterium*, and *Blautia*, and one OTU annotated to each of the species *Bifidobacterium longum, Bifidobacterium breve*, and *Faecalibaculum rodentium*.

**Figure 2:**
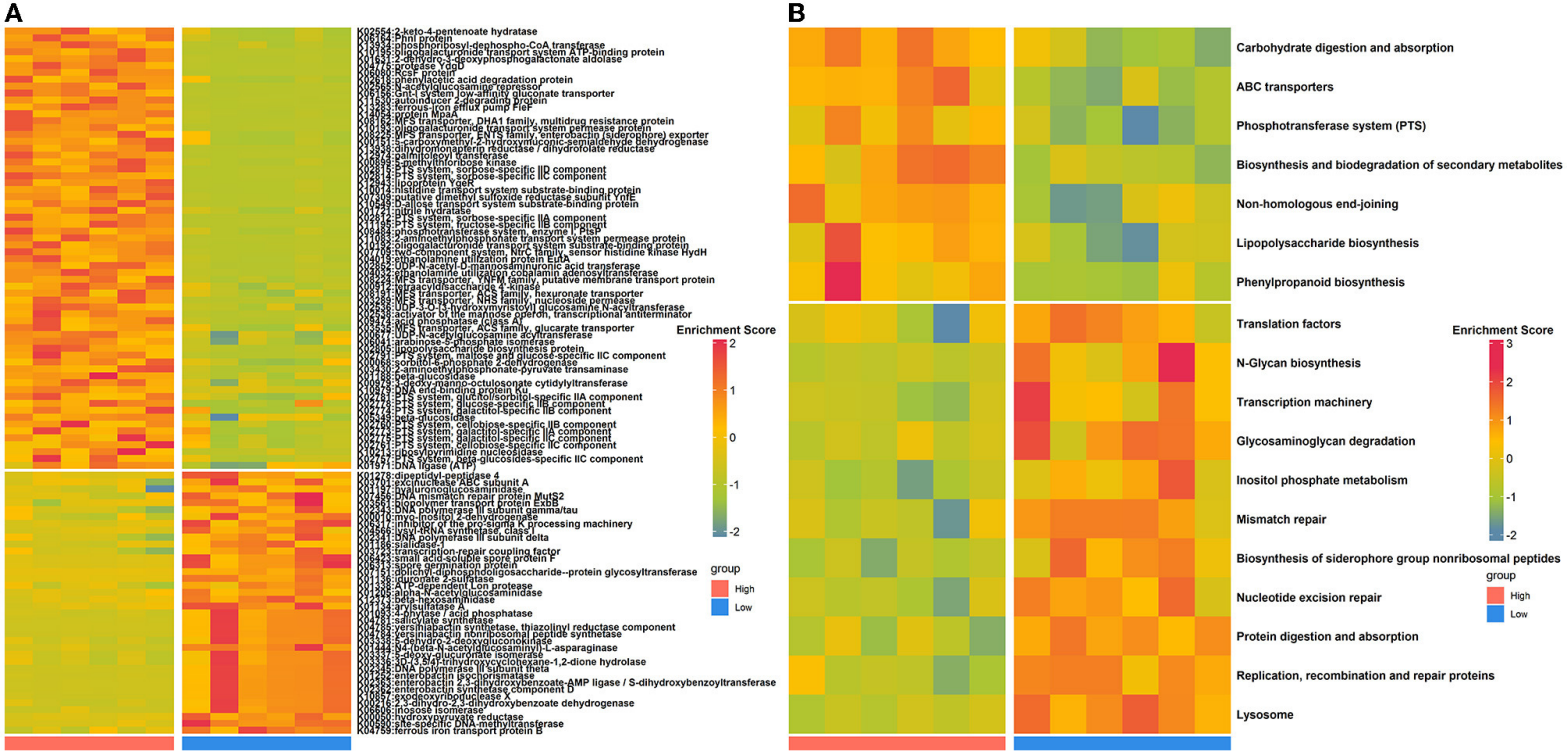
Differentially enriched nasal microbial KOs **(A)** and KEGG pathways **(B)** between high- and low-level house ammonia-exposed rabbits.

To evaluate how the nasal microbial functional capacities altered as house ammonia levels varied, the predicted KOs and KEGG pathways were compared between the two groups of rabbits (Figure 2; Supplementary Table S4). Sixty-four KOs were highly represented in rabbits exposed to high levels of house ammonia, of which most were assigned to ABC transporters, the phosphotransferase system (PTS), lipopolysaccharide biosynthesis, phenylpropanoid biosynthesis, and non-homologous end-joining. Moreover, 38 KOs were significantly enriched in rabbits exposed to low-level house ammonia, of which most were related to the lysosome, biosynthesis of siderophore group non-ribosomal peptides, inositol phosphate metabolism, mismatch repair, and nucleotide excision repair. KEGG pathway comparison analysis showed that seven functional categories, including ABC transporters, the phosphotransferase system (PTS), and lipopolysaccharide biosynthesis, were more prevalent in rabbits exposed to high levels of house ammonia. In addition, 11 functional categories, such as the lysosome, nucleotide excision repair, biosynthesis of siderophore group non-ribosomal peptides, mismatch repair, and inositol phosphate metabolism, were more active in rabbits exposed to low-level household ammonia.

### House ammonia impacts the colonic microbiota of rabbits

Regarding the alpha diversity, the Shannon and ACE indices significantly declined in rabbits exposed to an increasing level of house ammonia (Supplementary Figures S3A, B). Additionally, hierarchical clustering analysis of UPGMA indicated that the colonic microbiota of rabbits exposed to high- and low-level house ammonia exhibited a clear separation (Supplementary Figures S3C, D).

**Figure 3:**
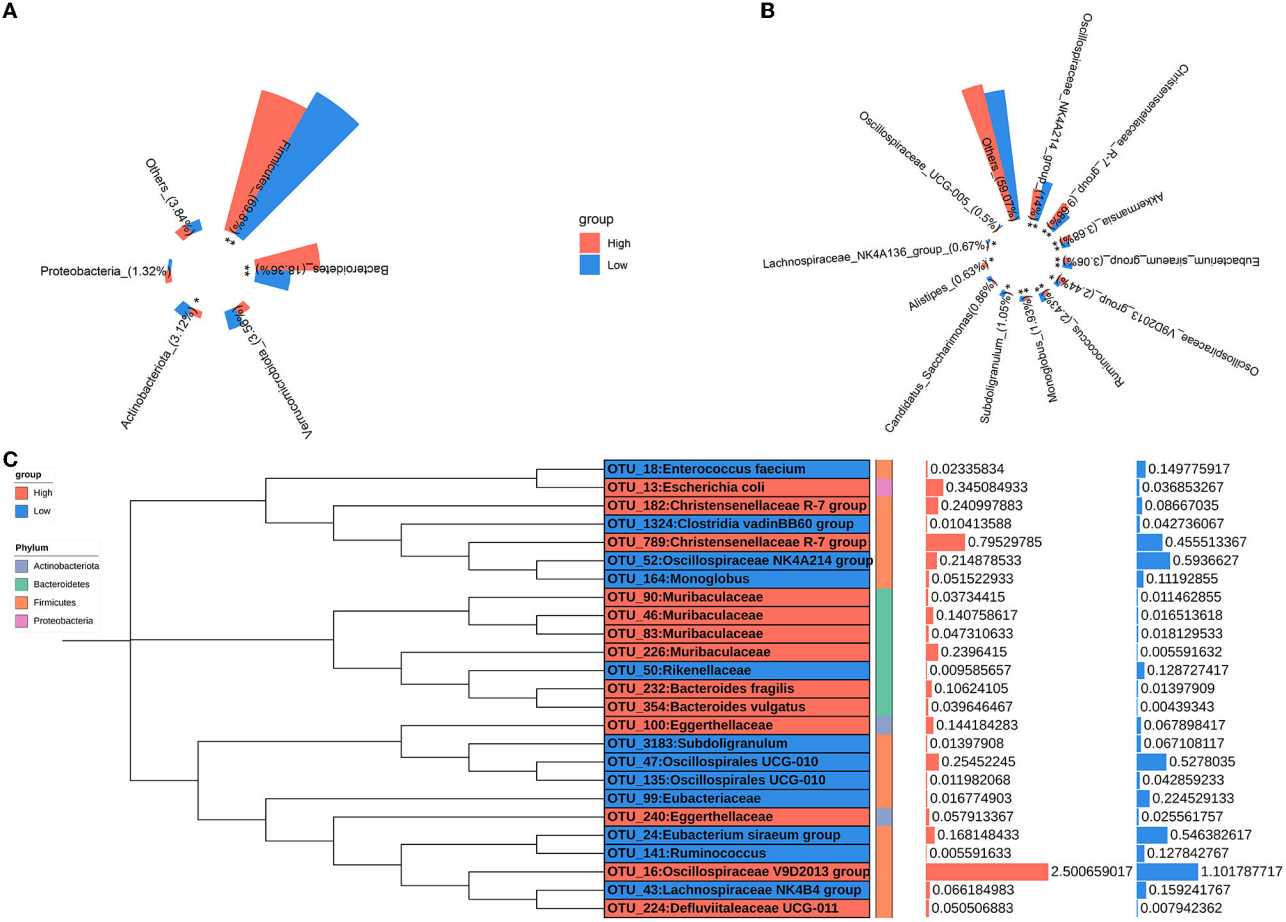
Changes in the relative abundances of colonic microbial phyla **(A)**, genera **(B)**, and OTUs **(C)**. “*”, FDR-adjusted *P* < 0.05, “**”, FDR-adjusted *P* < 0.01.

The relative abundances of microbial taxa were assessed to investigate the alterations in colonic microbial compositions. At the phylum level, *Firmicutes, Bacteroidetes, Verrucomicrobiota, Actinobacteriota*, and *Proteobacteria* were predominant in all samples, comprising more than 95% of the total reads (Figure 3A). Compared to the low-level house ammonia-challenged rabbits, the relative abundances of *Firmicutes* and *Actinobacteriota* in the high-level house ammonia-challenged rabbits were remarkably decreased, but the relative abundance of *Bacteroidetes* was significantly increased. At the genus level, the top 12 genera accounted for ~50% of total reads (Figure 3B). While comparing the rabbits exposed to low concentrations of house ammonia with those exposed to high concentrations, it was observed that the latter had increased abundances of the *Christensenellaceae R-7* group, *Akkermansia, Oscillospiraceae V9D2013* group, and *Alistipes*, and decreased abundances of the *Oscillospiraceae NK4A214* group, *Eubacterium siraeum* group, *Ruminococcus, Subdoligranulum*, and *Lachnospiraceae NK4A136* group. As shown in Figure 3C, 13 OTUs showed higher abundances in rabbits exposed to a high concentration of house ammonia, including four OTUs classified as *Muribaculaceae*, two classified as *Christensenellaceae R-7* group, two as *Eggerthellaceae*, and one each classified as *Escherichia coli, Bacteroides fragilis, Bacteroides vulgatus, Defluviitaleaceae UCG-011*, and *Oscillospiraceae V9D2013* group. Moreover, 12 OTUs possessed greater proportions in rabbits exposed to low-level house ammonia, including two OTUs classified as Oscillospirales_UCG-010 and one each classified as *Enterococcus faecium, Eubacteriaceae, Lachnospiraceae NK4B4* group, *Oscillospiraceae NK4A214* group, *Clostridia vadinBB60* group, *Eubacterium siraeum* group, Rikenellaceae, *Subdoligranulum, Ruminococcus*, and *Monoglobus*.

**Figure 4:**
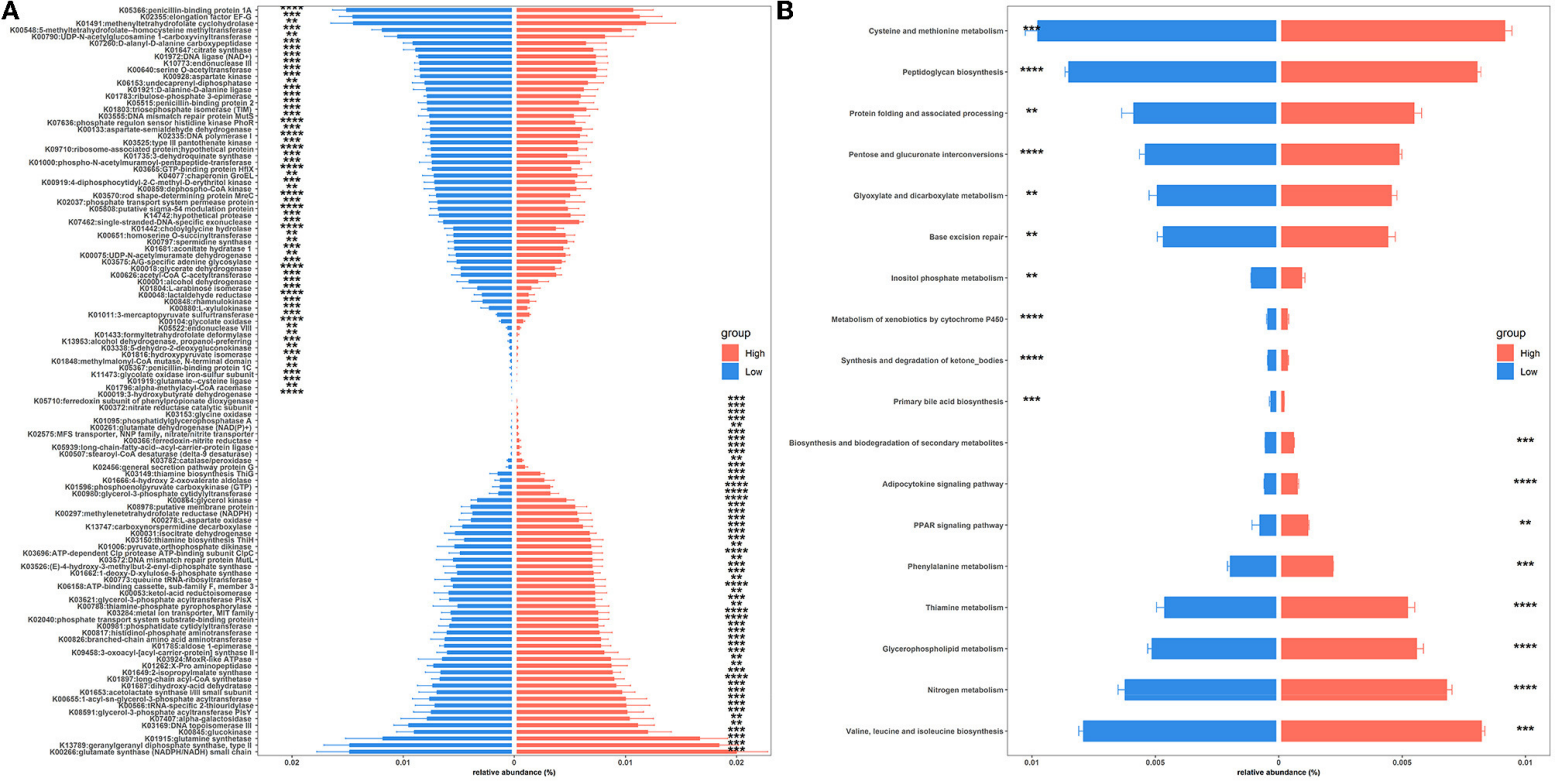
Significantly enriched colonic microbial KOs **(A)** and KEGG pathways **(B)** between the high and low concentrations of house ammonia-challenged rabbits. “**”, FDR-adjusted *P* < 0.01, “***”, FDR-adjusted *P* < 0.005, “****”, FDR-adjusted *P* < 0.001.

To gain insights into functional alterations of the colonic microbiota, the predicted gene catalog was aligned with the KEGG database. A total of 113 KOs were significantly different in abundance between high- and low-level house ammonia-challenged rabbits (Figure 4A; Supplementary Table S5). The KOs were more abundant in rabbits exposed to a high concentration of house ammonia related to nitrogen metabolism, phenylalanine metabolism, valine, leucine and isoleucine biosynthesis, glycerophospholipid metabolism, and thiamine metabolism. The KOs showed higher abundance in rabbits exposed to low concentrations of house ammonia and were associated with peptidoglycan biosynthesis, cysteine and methionine metabolism, glyoxylate and dicarboxylate metabolism, base excision repair, and pentose and glucuronate interconversions. However, 18 KEGG pathways manifested significantly different abundances between the two groups of rabbits (Figure 4B). Similar to KO analysis results, valine, leucine and isoleucine biosynthesis, nitrogen metabolism, glycerophospholipid metabolism, thiamine metabolism, and phenylalanine metabolism were more abundant in rabbits exposed to high concentrations of house ammonia, while cysteine and methionine metabolism, peptidoglycan biosynthesis, pentose and glucuronate interconversions, glyoxylate and dicarboxylate metabolism, and base excision repair were enhanced in rabbits exposed to low concentrations of household ammonia.

### House ammonia influences the lung and colon transcriptomes of rabbits

**Figure 5:**
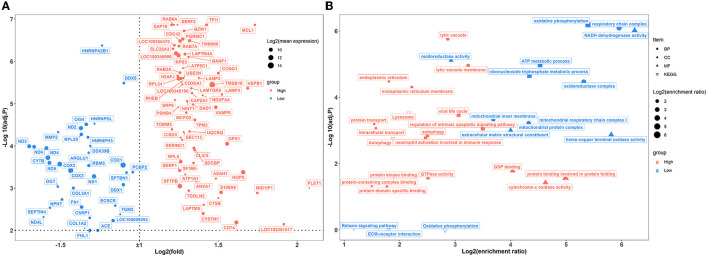
Transcriptome sequencing analysis of lung samples under different levels house ammonia exposure. **(A)** Differentially expressed genes. **(B)** Functional enrichment analysis of differentially expressed genes.

A total of 100 pulmonary differentially expressed genes (DEGs) were identified between the two groups of rabbits (Figure 5A). GO enrichment analysis showed that DEGs were enriched in nine categories of biological processes, nine categories of cellular components, and 11 categories of molecular function (Figure 5B; Supplementary Table S6). Among these, DEGs showed greater expression levels in rabbits exposed to a high concentration of house ammonia and are mainly involved in the regulation of the intrinsic apoptotic signaling pathway, autophagy, lytic vacuole, viral life cycle, neutrophil activation involved in immune response, and cytochrome-c oxidase activity. Additionally, upregulated DEGs in rabbits exposed to low concentrations of ammonia mainly contributed to oxidative phosphorylation, oxidoreductase complex, and NADH dehydrogenase activity. Similar results were observed in the KEGG enrichment analysis (Figure 5B). The DEGs derived from rabbits exposed to a high concentration of house ammonia engaged in lysosome and autophagy functions, while the DEGs from the rabbits exposed to a low concentration of house ammonia participated in oxidative phosphorylation, ECM-receptor interaction, and the relaxin signaling pathway.

**Figure 6:**
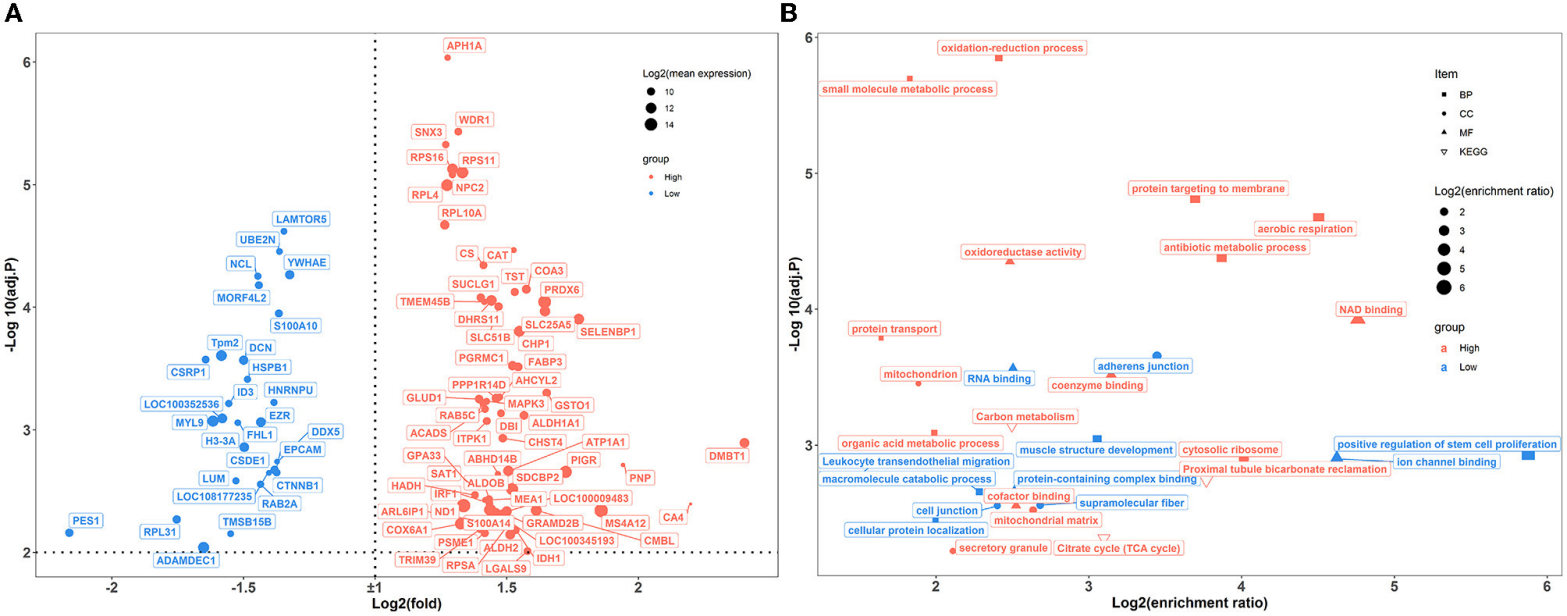
Transcriptome sequencing analysis of colon samples challenged by different concentrations of house ammonia. **(A)** Differentially expressed genes. **(B)** Functional enrichment analysis of differentially expressed genes.

In the colon, 89 genes were differentially expressed between the two groups of rabbits (Figure 6A). A total of 25 enriched GO functional terms were identified, including 11 terms of biological processes, seven terms of cellular components, and eight terms of molecular function (Figure 6B; Supplementary Table S7). Among them, the highly expressed DEGs of high-level house ammonia-challenged rabbits related to aerobic respiration, antibiotic metabolic process, oxidation-reduction process, cytosolic ribosome, and NAD binding. In addition, the enhanced DEGs of rabbits exposed to low-level house ammonia were associated with positive regulation of muscle structure development, adherens junctions, and ion channel binding. Furthermore, KEGG enrichment analysis indicated that DEGs were mainly enriched in carbon metabolism, proximal tubule bicarbonate reclamation, citrate cycle, and leukocyte transendothelial migration (Figure 6B).

### House ammonia perturbs the muscle metabolome of rabbits

A total of 515 metabolites were identified using UPLC-QTOFMS. Multivariate statistical analyses, including PCA and OPLS-DA, were performed to evaluate the variations in metabolic patterns between the two groups of rabbits. The results showed that high- and low-level house ammonia-challenged rabbits were clustered separately, which implied distinctive metabolic patterns (Supplementary Figure S4).

**Figure 7:**
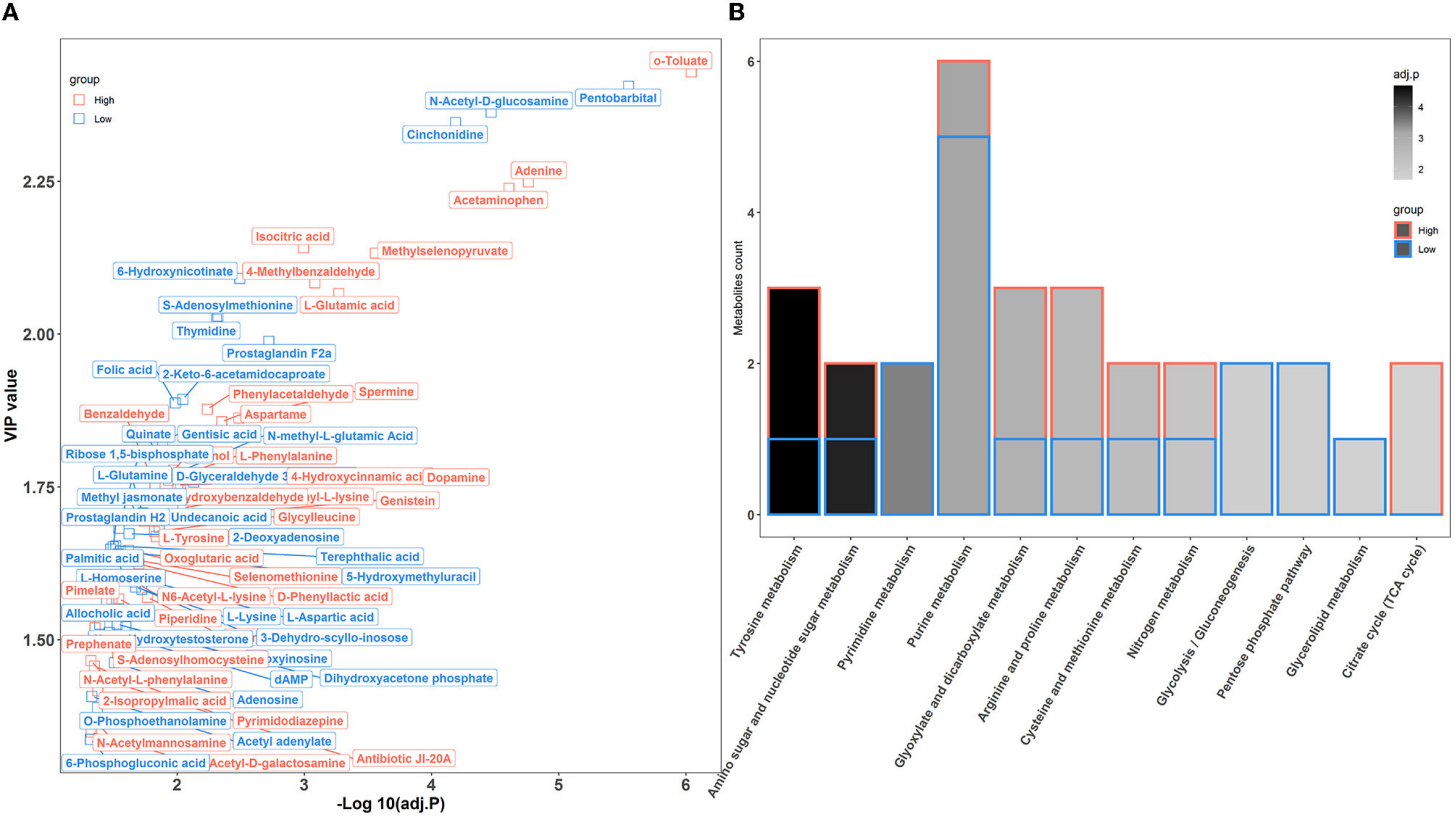
Muscle metabolic profiling analysis in rabbits exposed to different levels of house ammonia. **(A)** Differentially metabolites. **(B)** KEGG enrichment analysis of differential metabolites.

To further confirm the metabolic disruption effect of house ammonia, univariate analysis was performed on each metabolite. As shown in Figure 7A, 70 metabolites were significantly affected by different levels of house ammonia exposure. Among them, 35 differentially abundant metabolites were enriched in rabbits exposed to high-level house ammonia exposure, with 10 amino acids and their derivatives (e.g., L-glutamic acid, beta-alanyl-L-lysine, L-phenylalanine, L-tyrosine, and L-ornithine), eight organic acids and their derivatives (e.g., o-toluate, methylselenopyruvate, isocitric acid, and oxoglutaric acid), three phenols (acetaminophen, dopamine, and phenol), three benzoyl derivatives (4-methylbenzaldehyde, phenylacetaldehyde, and benzaldehyde), three organic oxygen compounds (*N*-acetylmannosamine, N-acetyl-D-galactosamine, and 4-hydroxybenzaldehyde), and two fatty acids and derivatives (pimelate and 2-Isopropylmalic acid). The remaining 35 differentially abundant metabolites were concentrated in rabbits exposed to low concentrations of house ammonia, with seven fatty acids and their derivatives (e.g., prostaglandin F2a, prostaglandin H2, palmitic acid, and alcoholic acid), seven organic oxygen compounds (e.g., *N*-acetyl-D-glucosamine, D-glyceraldehyde 3-phosphate, quinate, and dihydroxyacetone phosphate), six amino acids and their derivatives (e.g., L-aspartic acid, L-glutamine, and L-lysine), five organic acids and their derivatives (e.g., 2-Keto-6-acetamidocaproate, folic acid, and gentisic acid), and three pyrimidines and their derivatives (e.g., pentobarbital, thymidine, and 5-hydroxymethyluracil). KEGG enrichment analysis indicated that differentially abundant metabolites are involved in 12 metabolic processes (Figure 7B). It is intriguing to note that tyrosine metabolism, glyoxylate, and dicarboxylate metabolism, arginine and proline metabolism, and the citrate cycle (TCA cycle) were significantly increased in high-level house ammonia-challenged rabbits, while pyrimidine metabolism, purine metabolism, glycolysis/gluconeogenesis, the pentose phosphate pathway, and glycerolipid metabolism were highly active in low-level house ammonia-challenged rabbits.

### Integrated analysis of multi-omics data

**Figure 8:**
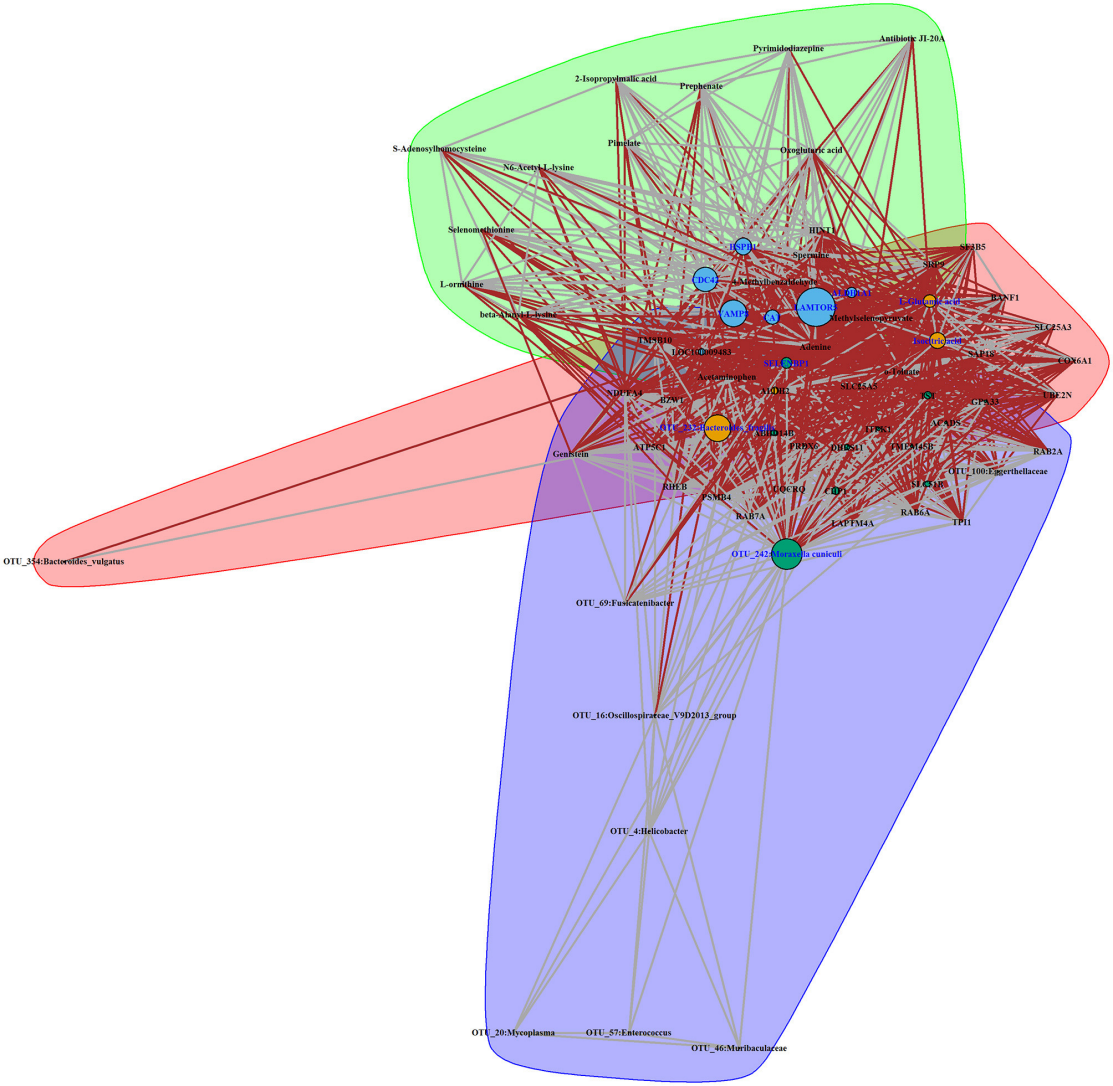
Interaction network of features identified in a high concentration of house ammonia-challenged rabbits. Each node represents a feature, and each edge denotes a significant interaction. The highlighted clusters, nodes, and edges stand for communities, key features, and inter-community interactions, respectively.

To explore the systematically impairing action of house ammonia on rabbits, a correlation network was first generated based on potential biomarkers identified in each omic dataset of high-level house ammonia-challenged rabbits (Supplementary Figure S5), and then, the features in the core community in each network were selected for constructing the integrated network. Finally, three main communities containing 70 interrelated features were detected (Figure 8), which suggested statistically robust interactions within and between each omic dataset. Importantly, 11 key features were identified in the integrated network. Among these, six features were presented in the light green community, including four lung genes (*LAMTOR5, VAMP8, CDC42*, and *HSPB1*) and two colon genes (*CAT* and *ALDH1A1*). Three features, including two muscle metabolites (L-glutamic acid and isocitric acid) and one colonic microbe (OTU_232:*Bacteroides fragilis*), were exhibited in the coral-red community. The colon gene SELENBP1 and the nasal microbe OTU_242:*Moraxella cuniculi* were recognized in the violet community.

**Figure 9:**
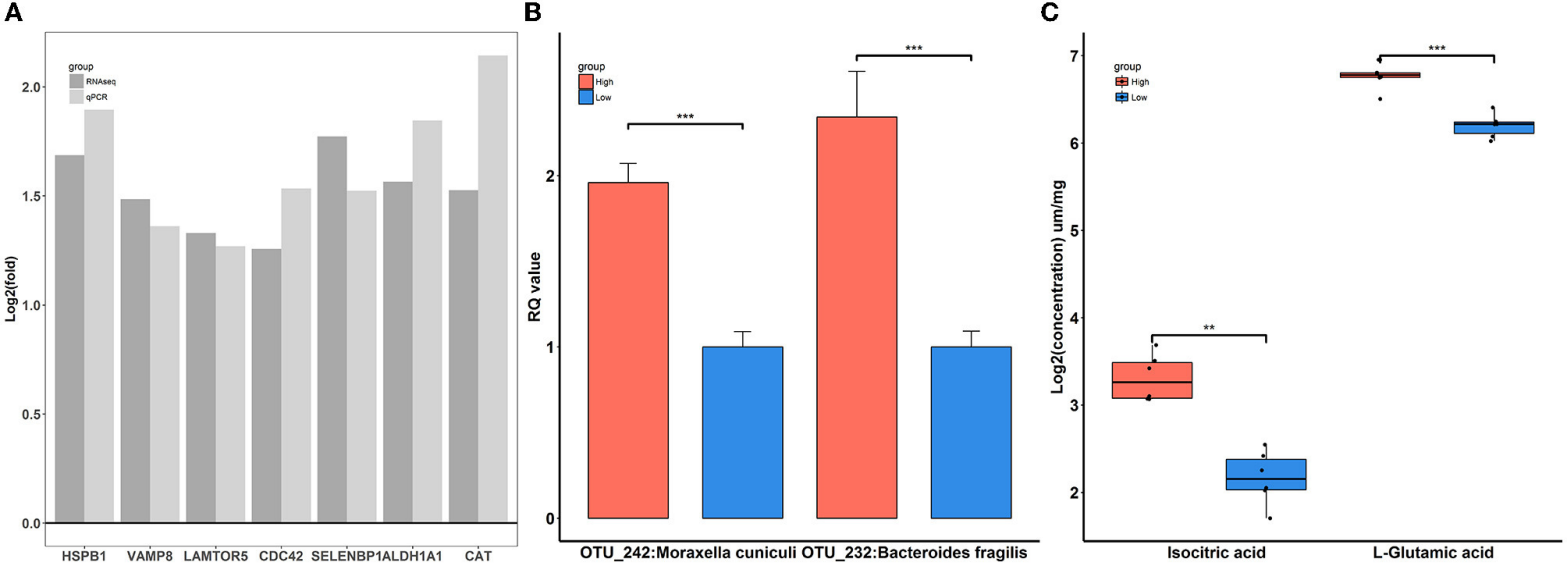
Verification of differentially expressed genes **(A)**, significantly enriched microbes **(B)**, and differential metabolites **(C)**. “^**^”, FDR adjust *P* < 0.01, “^***^”, FDR adjust *P* < 0.005.

To confirm the reliability of multi-omics data, key features were used for qPCR validation and quantitative metabolite assays. As shown in Figure 9A, the gene expression patterns of *HSPB1, VAMP8, LAMTOR5, CDC42, SELENBP1, ALDH1A1*, and *CAT* between RNA-Seq and qPCR were similar. Compared to rabbits exposed to low-level house ammonia, the OTU_242:*Moraxella cuniculi* and OTU_232:*Bacteroides fragilis* showed markedly increased abundances in rabbits exposed to high-level house ammonia, which is in line with the results of 16S rRNA gene sequencing analysis (Figure 9B). Additionally, the alterations in isocitric acid and L-glutamic acid between the two groups of rabbits were in accordance with muscle metabolic profile characterization (Figure 9C).

## Discussion

Ammonia emissions are a major concern in intensive livestock production systems. It not only adversely affects the ecosystem and environment but is also harmful to the health of both animals and humans. Although previous studies revealed the detrimental effects of ammonia exposure on different farm animals, most conclusions were drawn based on environment control chambers, which may fail to reflect the complicated ambient conditions of barns. In the current study, multi-omics data composed of the microbiome, transcriptome, and metabolome were analyzed to systematically and comprehensively understand the deleterious effects of house ammonia on rabbits reared in a commercial, confined barn.

### Ammonia exposure causes nasal microbiota alterations

The nose is the major interface between the internal body and the external environment, and tremendous changes in the porcine nasal microbial community have been observed following different concentrations of house ammonia exposure (Wang et al., 2019). Similarly, we found that the nasal microbiota of rabbits was notably affected by different levels of house ammonia treatment. A remarkable reduction in nasal microbial alpha diversity was observed when rabbits were exposed to high levels of house ammonia, and beta diversity also exhibited significant differences between high- and low-level house ammonia-exposed rabbits (Supplementary Figure S1). Nasal cavity pH is regarded as an important factor that affects microbiota colonization, while ammonia exposure disrupts acid-base equilibrium and may result in nasal microbial biodiversity alterations (Morawska-Kochman et al., 2019).

In accordance with previous studies on nasal microbial phylogenetic composition (Qin et al., 2019; Gomez et al., 2021), our results indicated that *Proteobacteria, Firmicutes*, and *Bacteroidetes* were the most prevalent phyla in the nasal microbial community. Moreover, rabbits exposed to high levels of house ammonia presented a noticeable augmentation of *Proteobacteria* and a depletion of *Firmicutes* and *Bacteroidetes* (Figure 1A). *Proteobacteria* comprises several well-known opportunistic pathogens (e.g., *Pasteurella, Neisseria*, and *Campylobacter*), which showed increased abundance in conjunction with losses in *Firmicutes* and *Bacteroidetes*, as reported in the nasal microbial communities of humans with chronic rhinosinusitis and pigs with Glässer's disease (Choi et al., 2014; Correa-Fiz et al., 2016). This suggests that house ammonia exposure leads to variations in these predominant phyla in the nasal microbiota and may increase rabbits' susceptibility to respiratory tract infections and diseases. We also identified 20 abundant genera that accounted for over 85% of the total sequences (Figure 1B). Among these, it is worth noting that the relative abundance of *Moraxella, Escherichia-Shigella*, and *Mannheimia* significantly increased in high-level house ammonia-challenged rabbits, while *Akkermansia, Lactobacillus*, and *Bifidobacterium* distinctly declined. Although previous studies demonstrated that *Moraxella* was commonly present in the nasal microbiota of animals and humans, its overgrowth was associated with an increased risk of respiratory illness due to its potential role in inducing epithelial damage and inflammatory cytokine expression (McCauley et al., 2019; Lopez-Serrano et al., 2020). This should be due to the synergistic effect of house ammonia exposure on nasal mucosal injuries, possibly promoting the proliferation of *Moraxella* and aggravating inflammatory responses (Urbain et al., 1996). In addition, nasal microbiota profiles dominated by both *Escherichia-Shigella* and *Mannheimia* were previously reported to have intimate relationships with respiratory disorders in broilers and steers (Cirone et al., 2019; Liu et al., 2020).

However, *Akkermansia, Lactobacillus*, and *Bifidobacterium* could regulate epithelial barrier function and immune response, and their absences were closely correlated with decreased respiratory tract immunity (Heintz-Buschart et al., 2018; Kim et al., 2019; Wang et al., 2019). Importantly, we found that several distinctly different OTUs annotated to the specific species belonged to the abovementioned genera, for example, OTU_21:*Escherichia coli*, OTU_242:*Moraxella cuniculi*, OTU_29:*Bifidobacterium longum*, and OTU_574:*Bifidobacterium breve* (Figure 1C). This implies that certain nasal microbial species should be regarded as potential indicators of house ammonia exposure detrimental to respiratory health in rabbits.

We also assessed the functional variations of the nasal microbiota under different levels of house ammonia exposure (Figure 2). As the level of exposure to house ammonia increased, functional processes, such as ABC transporters and lipopolysaccharide biosynthesis, which are capable of driving respiratory tract-cascaded inflammatory responses, were activated (Lee et al., 2019; Ma et al., 2021). Moreover, functional items that have regulatory roles in pathogens' invasion defense and epithelial cell injury repair, such as the lysosome and inositol phosphate metabolism, were inhibited (Cohen et al., 2016; Wu S. E. et al., 2020). These results suggested that a high concentration of house ammonia exposure may mediate the respiratory inflammatory processes by disturbing the nasal microbial functional homeostasis.

### Ammonia exposure induces colonic microbial changes

The symbiotic relationship between the host and gut microbiota ensures the appropriate development of the metabolic and immune systems, which is crucial for host health (Jandhyala et al., 2015). However, a growing body of evidence suggests that the inhalation of excessive ammonia causes gut microbial dysbiosis, which consequently influences the health and growth of farm animals (Li et al., 2021b; Zhou et al., 2021). Our results also showed that the colonic microbial communities of rabbits were continuously changed following house ammonia exposure. Gut microbial alpha diversity was notably decreased with exposure to an increased level of house ammonia, and beta diversity was dramatically altered under different concentrations of house ammonia (Supplementary Figure S2), which confirmed that ammonia exposure caused gut microbial dysbiosis in animals (Tao et al., 2019; Han H. et al., 2021).

The colonic microbial composition also exhibited noticeable variations under high-level exposure to house ammonia (Figure 3). At the phylum level, ammonia exposure increased the relative abundance of *Bacteroidetes* and decreased the relative abundance of *Firmicutes* and *Actinobacteriota*. This could be due to ammonia exposure promoting the production of urea that flows into the colon, which facilitates the preferential proliferation of urea metabolism bacteria derived from *Bacteroidetes* (Tang et al., 2020; Yin et al., 2022). Consistently, we found that the growth of the genus *Alistipes*, which belongs to the phylum *Bacteroidetes*, was promoted following high-level house ammonia exposure, while the growth of genera in the phylum Firmicutes, such as the *Eubacterium siraeum* group, *Ruminococcus*, and *Subdoligranulum*, was inhibited. Although *Alistipes* are commonly present in a relatively low proportion, it is highly relevant to intestinal dysbiosis and inflammation in humans and animals. For instance, the augmentation of *Alistipes* and the elevation of lipopolysaccharide biosynthesis were intimately associated with intestinal inflammation and permeability in hypertension patients (Kim et al., 2018). A metagenomic study in a mouse model of ileitis suggested that *Alistipes* showed a strong correlation with ileitis incidence (Rodriguez-Palacios et al., 2018).

In contrast, the *Eubacterium siraeum* group, *Ruminococcus*, and *Subdoligranulum* are butyric acid-producing bacteria that have beneficial effects on maintaining intestinal epithelial integrity and immune functions, and their elimination in inflammatory conditions will aggravate intestinal impairment (Zhu et al., 2020; Correa et al., 2021). Additionally, we noticed that the potentially beneficial bacteria *Akkermansia* was enriched by a high concentration of house ammonia exposure, which was different from the observation in the nasal microbiota. *Akkermansia* in the colon may act as scavengers, which clean the mucin produced by ammonia exposure (Duan et al., 2018).

Nonetheless, *Akkermansia* thrives in the gut microbiota and may be capable of inducing the incidence of epizootic rabbit enteropathy (Jin et al., 2018). At the OTU level, we further identified several important microbial species related to house ammonia exposure, such as *Bacteroides fragilis* (OTU232), *Bacteroides vulgatus* (OTU354), and *Enterococcus faecium* (OTU18). As mentioned above, a high concentration of ammonia exposure led to an increase in colonic urea flux, and urea was eventually broken down into endogenous ammonia with the aid of bacterial urease. Coincidentally, *Bacteroides vulgatus* is an ureolytic bacterium, and *Bacteroides fragilis* can utilize endogenous ammonia as a primary nitrogen source to promote its growth and reproduction (Forsythe and Parker, 1985; Gibson and Macfarlane, 1988). However, a high proportion of *Bacteroides fragilis* is implicated in immune system dysfunction and colonic inflammatory conditions (Thiele Orberg et al., 2017; Chung et al., 2018). Moreover, the depletion of *Enterococcus faecium* caused by ammonia exposure has a negative impact on intestinal epithelial cell barrier function, increasing ammonia toxicity (Park et al., 2016).

In agreement with the results of recent studies on functional alterations of intestinal microbiota under ammonia exposure (Duan et al., 2021; Tang et al., 2021b), our functional capacity analysis demonstrated that colonic microbiota were more engaged in amino acid metabolism (e.g., valine, leucine, and isoleucine biosynthesis and phenylalanine metabolism) and lipid metabolism (e.g., glycerophospholipid metabolism) following high-level house ammonia exposure but less engaged in carbohydrate metabolism (e.g., peptidoglycan biosynthesis, pentose and glucuronate interconversions, and glyoxylate and dicarboxylate metabolism). A low intestinal pH and the presence of carbohydrates restrict amino acid metabolism (Louis and Flint, 2017), but increased pH caused by ammonia exposure could remove this inhibition. Furthermore, *in vitro* experiments indicated that microbes could modulate amino acid and lipid metabolic strategies to respond to environmental stress, which may help explain our observations (Stancik et al., 2002; Li et al., 2019).

### Ammonia exposure modulates pulmonary and colonic gene expression

Several studies have reported that ammonia exposure could regulate gene expression patterns in different tissues of farm animals (Wang et al., 2020; Li et al., 2021a). In the lung, our transcriptome sequencing results showed that house ammonia exposure led to remarkable alterations in gene expressions related to cell death (e.g., regulation of intrinsic apoptotic signaling pathway, autophagy, and lytic vacuole) and immune response (e.g., viral life cycle and neutrophil activation involved in the immune response) (Figure 5).

Cell death is an important modulator in response to a variety of environmental and internal stressors (Martins et al., 2011). We have identified several DEGs, including *MCL1, TMBIM6, HSPB1*, and *CD74*, that are associated with cell death processes. Myeloid cell leukemia 1 (*MCL1*) is a unique Bcl-2 family member with a significantly short half-life but an important anti-apoptotic function (Wu X. et al., 2020). A recent broiler study indicated that ammonia exposure can cause lung injury, which may be due to the increased expression level of *MCL1*, causing a delay in neutrophil apoptosis and leading to a disrupted immune response (Blomgran et al., 2012; Liu et al., 2020). Ammonia exposure has been implicated in inducing endoplasmic reticulum stress, and the activation of cell autophagy and the apoptosis-related protein transmembrane BAX inhibitor motif containing 6 (*TMBIM6*) play a regulatory role in response to endoplasmic reticulum stress (Han Q. et al., 2021; Kim et al., 2021). Heat shock protein beta 1 (*HSPB1*) is another cell autophagy and apoptosis-associated protein that commonly participates in different types of stress resistance, and its expression was upregulated in this study, which may be related to the cellular response to stress caused by house ammonia exposure (Wu et al., 2016; D'Anna et al., 2017). *CD74* binds to specific cytokines and exerts important roles in orchestrating a variety of pathological processes, such as cell necroptosis and the inflammatory response, and an elevated expression level of *CD74* is positively associated with lung injury (Takahashi et al., 2009; Su et al., 2017).

The precise and targeted surveillance mechanisms at the lung-environment interface are essential for maintaining pulmonary homeostasis and functions; however, inhaled environmental irritants can stimulate and disrupt the immune response of the lung, leading to potentially pathological consequences (Guttenberg et al., 2021). Upon ammonia stimulation, the immune response associated DEGs such as *CDC42, LAMTOR5, VAMP8*, and *CTSB* were identified. Cell division cycle 42 (*CDC42*) is a Rho GTPase that affects T-cell differentiation and inflammatory cytokine production, and is capable of initiating airway inflammation responses (Chen L. et al., 2021). Although the late endosomal/lysosomal adaptor and MAPK and MTOR activator 5 (*LAMTOR5*) are characterized as indispensable components of the amino acid sensing machinery, their key role in modulating lung immunosuppression has recently been noted (Wang L. et al., 2021). Here, the upregulation of *CDC42* and *LAMTOR5* expression may be correlated with immune dysfunctions of the airways caused by ammonia exposure (Chen J. et al., 2021; Wang H. et al., 2021). Vesicle-associated membrane protein 8 (*VAMP8*) can be involved in antigen cross-presentation and activation of cytolytic T-cell immune responses (Dingjan et al., 2017). Cystatin B (*CSTB*) can modulate the immune response under pathological conditions by inhibiting cysteine proteases (Premachandra et al., 2013). Moreover, the increased expressions of both *CTSB* and *VAMP8* are associated with inflammatory and bacterial infection processes (Xia et al., 2019), which may indirectly reflect the dysbiosis of pulmonary immunity and microbiota triggered by ammonia exposure.

However, house ammonia exposure resulted in changes in colonic gene expression that were related to the redox state (e.g., oxidation-reduction process, aerobic respiration, oxidoreductase activity, and NAD binding) (Figure 6). Most environmental pollutants could activate the antioxidant defense system, thereby affecting the redox state (Zheng et al., 2020). This study presented redox state-related DEGs such as CAT, SELENBP1, GLUD1, and ALDH1A1. Catalase (*CAT*) is an important member of the antioxidant defense system that plays a vital role in maintaining redox balance. In line with our findings, the expression level of *CAT* in aquatic animals also increases with excessive exposure to ammonia (Sun et al., 2014; Hongxing et al., 2021). This can likely be attributed to the surplus production of reactive oxygen species (ROS) that occurs at high concentrations of ammonia, leading to the upregulation of *CAT* expression (Zhang et al., 2008). Selenium binding protein 1 (*SELENBP1*) has been identified as a methanethiol oxidase in the colonic enterocytes, which catalyzes the conversion of methanethiol to redox signaling molecules hydrogen sulfide and hydrogen peroxide that engage in environmental stress responses (Sies and Jones, 2020; Philipp et al., 2021). In this study, the house ammonia challenge promotes the expression of *SELENBP1*, which may be linked to redox signaling molecule generation and transduction. Increased activity of glutamate dehydrogenase 1 (*GLUD1*) upon ammonia exposure has been observed in previous studies (Voss et al., 2021). It plays an important role in catalyzing the conversion of glutamate to alpha-ketoglutarate, which is known to maintain redox homeostasis and serve as a substrate for the detoxification of ammonia (Jin et al., 2015). Aldehyde dehydrogenase A1 (*ALDHA1*) catalyzes the detoxification of toxic unsaturated aldehydes generated by lipid peroxidation during redox imbalance (Calleja et al., 2021). It is well-recognized that ammonia exposure could trigger lipid peroxidation to produce malondialdehyde (Li and Qi, 2019), which may further induce the expression of *ALDHA1*.

### Ammonia exposure interferes with the muscular metabolic profile

Previous studies have demonstrated that ammonia exposure results in perturbations of amino acids, nucleotides, energy, and lipid metabolism in animals (Dong et al., 2020; Tang et al., 2020; Qin et al., 2022). We have consistently found that tyrosine metabolism, arginine and proline metabolism, pyrimidine metabolism, purine metabolism, citrate cycle (TCA cycle), glycolysis/gluconeogenesis, pentose phosphate pathway, and glycerolipid metabolism were influenced by house ammonia exposure (Figure 7). More importantly, we identified a variety of differentially abundant metabolites that should be regarded as signals for metabolic alterations under ammonia exposure. Glutamate (L-glutamic acid) is a key player in ammonia removal in a muscle, capturing ammonia to form glutamine. Glutamine is the major inter-organ carrier of ammonia that can transport ammonia in the muscle to the liver for detoxification (Hakvoort et al., 2017). However, high-level ammonia exposure could cause the initial ammonia condensation reaction to be less efficient (Dong et al., 2020). In addition, glutamine also serves as an important precursor for purine and pyrimidine synthesis, and the enhanced detoxification of ammonia could reduce nitrogen donors for nucleotide synthesis (Cory and Cory, 2006). Hence, glutamate accumulation, glutamine consumption, and nucleotide anabolism depletion were observed in this study. Notably, glutamate, as a central junction for the interchange of amino nitrogen, could facilitate L-ornithine synthesis (Blachier et al., 2009). L-ornithine participates in the urea cycle, leading to the excretion of excess ammonia and the generation of L-arginine and L-proline (Hoche et al., 2004). In addition, ammonia is utilized as a nitrogen source by aminotransferases for the *de novo* synthesis of aromatic amino acids such as L-tyrosine and L-phenylalanine, which should be another effective way to reduce ammonia content in muscle (Wang S. et al., 2021). Therefore, the metabolic activities of tyrosine, arginine, and proline were also enhanced.

However, energy metabolism processes such as the TCA cycle and glycolysis were impaired by a high concentration of house ammonia exposure. This could be partially explained by ammonia being used as a substrate for catalyzing the α-ketoglutarate (oxoglutaric acid) to glutamate conversion by glutamate dehydrogenase in muscle mitochondria and high-level ammonia stimulation enhancing the drain of α-ketoglutarate from the TCA cycle adversely affects ATP generation (Davuluri et al., 2016). Additionally, isocitrate dehydrogenase has been reported to be inhibited by ammonia, resulting in the accumulation of isocitrate (isocitric acid), which could further interfere with the TCA cycle (Katunuma et al., 1966). The impairment of the TCA cycle will cause a reduction in glycolysis due to feedback inhibition (Drews et al., 2020).

### Integrated analysis and validation experiment

To gain insights into the interactive relationships between different biological layers concerning distinct features linked to house ammonia exposure, an integrative correlation network was constructed based on multi-omics data (Figure 8). The network modeling yielded three communities composed of intertwined microbes, genes, and metabolites, indicating close intersystem interactions that may allow for proposing certain hypotheses to be tested in further studies focusing on house ammonia impairment mechanisms. For instance, there are close immune and metabolic interactions between the respiratory and gastrointestinal tracts, which are commonly referred to as the gut–lung axis, and the important roles of the gut–lung axis in pathophysiological processes caused by air pollution exposure have been generally recognized (Keulers et al., 2022; Mousavi et al., 2022). Moreover, the existence of a gut-muscle axis has been suggested more recently, and the gut–muscle axis is increasingly implicated in endogenous ammonia and urea nitrogen metabolism (Ticinesi et al., 2019; Yeh et al., 2022). Moreover, the specific roles of highlighted features such as OTU_242:*Moraxella cuniculi*, OTU_232:*Bacteroides fragilis, HSPB1, LAMTOR5, CDC42, CAT*, and L-glutamic acid present in the network communities had been discussed above, and their alterations under house ammonia exposure were confirmed by different experimental measurements (Figure 9). This implies that these features should not be underestimated in dissecting the underlying mechanisms linked to the deleterious effects of house ammonia on rabbits.

## Limitations

Our study has several limitations. The first is that the small sample size within the single rabbit population limited our ability to detect more taxonomic and functional features related to house ammonia exposure. Investigations of larger and more diverse rabbit populations will probably help to identify additional biomarkers. Due to the relatively limited taxonomic resolution of 16S rRNA sequencing, high-resolution shotgun metagenomic sequencing should be performed to uncover the specific microbial species associated with house ammonia exposure in future studies. In addition, our study was also limited to an episode of house ammonia exposure outcomes, and therefore, it would be particularly interesting to perform time series multi-omics analyses to capture the dynamic changes. Finally, *in vivo* experiments should be performed to explore how the cross-talk between different biological entities may exacerbate the detrimental roles of house ammonia exposure.

## Conclusions

In the present study, integrated analysis of the multi-omics data was performed to obtain overviews of changes in microbial communities, gene expressions, and metabolic profiles in rabbits exposed to house ammonia. The results indicated that house ammonia exposure caused dramatic variations in both nasal and intestinal microbial diversities, phylogenetic compositions, and functional capacities, which could potentially affect the immune responses and inflammatory processes of rabbits. Furthermore, house ammonia exposure led to genes being differentially expressed in the lung and colon and enriched functional terms related to cell death processes, immune responses, and redox state. A change in the muscular metabolic profile was also observed after house ammonia stimulation. Several crucial, differentially abundant metabolites, such as L-glutamic acid, L-glutamine, and L-ornithine, associated with amino acid and nucleotide metabolism, as well as oxoglutaric acid and isocitric acid, correlated with energy metabolism, were identified. Additionally, the widespread and strong inter-system cross-talk was outlined. Our findings provide valuable insights into the detrimental effects of house ammonia exposure on rabbits and pave the way for exploring the underlying impairment mechanisms.

## Data availability statement

The datasets presented in this study can be found in online repositories. The names of the repository/repositories and accession number(s) can be found below: https://db.cngb.org/-CNP0003860.

## Ethics statement

The animal study was reviewed and approved by Animal Care and Use Committee (ACUC) in Fujian Agriculture and Forestry University.

## Author contributions

SF conceived and designed the experiments, supervised the experiment progress, and wrote and revised the manuscript. QG designed the experiments, analyzed the data, and wrote and revised the manuscript. KL and SP performed the experiments, analyzed the data, and wrote the manuscript. ZL revised the manuscript. XD and YG performed the experiments. All authors contributed to the article and approved the submitted version.

## Supplementary material

Supplementary Figure S1 Diagram of confined barn structural parameters and air quality measurement instrument distribution. A, B, C, and D represent four sets of double-deck cages, which were further divided evenly into three parts (1, 2, and 3).

Supplementary Figure S2 House ammonia impacted the alpha **(A, B)** and beta **(C, D)** diversity of the nasal microbial community. “^***^,” FDR-adjusted *P* < 0.005.

Supplementary Figure S3 Colonic microbial alpha **(A, B)** and beta **(C, D)** diversity are altered by house ammonia. “^**^,” FDR-adjusted *P* < 0.01.

Supplementary Figure S4 PCA **(A)** and PLS-DA **(B)** analysis showing the effect of house ammonia on muscle metabolome.

Supplementary Figure S5 Network analysis of the interplay between nasal microbes **(A)**, colonic microbes **(B)**, lung genes **(C)**, colon genes **(D)**, and muscle metabolites **(E)** enriched in high-level house ammonia-exposed rabbits.

Supplementary Table S1 Air quality parameter measurements of high- and low-concentration ammonia-exposed rabbits.

Supplementary Table S2 Nutrients in the commercial pellet diet.

Supplementary Table S3 Primers used for qPCR analysis.

Supplementary Table S4 Significantly enriched nasal microbial KO pathway assignment.

Supplementary Table S5 Significantly enriched colonic microbial KO pathway assignment.

Supplementary Table S6 Pulmonary differentially expressed genes functional enrichment analysis.

Supplementary Table S7 Colonic differentially expressed genes functional enrichment analysis.

## References

[B1] Alvarado A. C. Predicala B. Z. (2019). Occupational exposure risk for swine workers in confined housing facilities. J. Agric. Saf. Health 25, 37–50. 10.13031/jash.12990 30893975

[B2] Blachier F. Boutry C. Bos C. Tome D. (2009). Metabolism and functions of L-glutamate in the epithelial cells of the small and large intestines. Am. J. Clin. Nutr. 90, 814S−21S. 10.3945/ajcn.2009.27462S 19571215

[B3] Blomgran R. Patcha Brodin V. Verma D. Bergstrom I. Soderkvist P. Sjowall C.. (2012). Common genetic variations in the Nalp3 inflammasome are associated with delayed apoptosis of human neutrophils. PLoS ONE 7, e31326. 10.1371/journal.pone.0031326 22403613 PMC3293864

[B4] Calleja L. F. Yoval-Sanchez B. Hernandez-Esquivel L. Gallardo-Perez J. C. Sosa-Garrocho M. Marin-Hernandez A.. (2021). Activation of Aldh1a1 by omeprazole reduces cell oxidative stress damage. FEBS J. 288, 4064–4080. 10.1111/febs.15698 33400378

[B5] Chen J. Jin A. Huang L. Zhao Y. Li Y. Zhang H.. (2021). Dynamic changes in lung microbiota of broilers in response to aging and ammonia stress. Front. Microbiol. 12, 696913. 10.3389/fmicb.2021.696913 34421851 PMC8371464

[B6] Chen L. Collado K. Rastogi D. (2021). Contribution of systemic and airway immune responses to pediatric obesity-related asthma. Paediatr. Respir. Rev. 37, 3–9. 10.1016/j.prrv.2020.02.005 32253127 PMC8477371

[B7] Chen S. Luo S. Yan C. (2021). Gut microbiota implications for health and welfare in farm animals: a review. Animals 12:93. 10.3390/ani12010093 35011199 PMC8749645

[B8] Choi E. B. Hong S. W. Kim D. K. Jeon S. G. Kim K. R. Cho S. H.. (2014). Decreased diversity of nasal microbiota and their secreted extracellular vesicles in patients with chronic rhinosinusitis based on a metagenomic analysis. Allergy 69, 517–526. 10.1111/all.12374 24611950

[B9] Chung L. Orberg E. T. Geis A. L. Chan J. L. Fu K. DeStefano Shields C. E.. (2018). Bacteroides fragilis toxin coordinates a pro-carcinogenic inflammatory cascade via targeting of colonic epithelial cells. Cell Host Microbe 23, 421. 10.1016/j.chom.2018.02.004 29544099 PMC6469393

[B10] Cirone F. Padalino B. Tullio D. Capozza P. Lo Surdo M. Lanave G.. (2019). Prevalence of pathogens related to bovine respiratory disease before and after transportation in beef steers: preliminary results. Animals. 9:1093. 10.3390/ani9121093 31817737 PMC6940923

[B11] Cohen T. S. Hilliard J. J. Jones-Nelson O. Keller A. E. O'Day T. Tkaczyk C.. (2016). *Staphylococcus aureus* alpha toxin potentiates opportunistic bacterial lung infections. Sci. Transl. Med. 8, 329ra.31. 10.1126/scitranslmed.aad9922 26962155

[B12] Correa P. S. Jimenez C. R. Mendes L. W. Rymer C. Ray P. Gerdes L.. (2021). Taxonomy and functional diversity in the fecal microbiome of beef cattle reared in Brazilian traditional and semi-intensive production systems. Front. Microbiol. 12, 768480. 10.3389/fmicb.2021.768480 34956130 PMC8692951

[B13] Correa-Fiz F. Fraile L. Aragon V. (2016). Piglet nasal microbiota at weaning may influence the development of glasser's disease during the rearing period. BMC Genom. 17, 404. 10.1186/s12864-016-2700-8 27230662 PMC4881051

[B14] Cory J. G. Cory A. H. (2006). Critical roles of glutamine as nitrogen donors in purine and pyrimidine nucleotide synthesis: asparaginase treatment in childhood acute lymphoblastic leukemia. In Vivo 20, 587–589. 17091764

[B15] Cui J. Wu F. Yang X. Liu S. Han S. Chen B.. (2021). Effects of ammonia on hypothalamic-pituitary-ovarian axis in female rabbits. Ecotoxicol. Environ. Saf. 227, 112922. 10.1016/j.ecoenv.2021.112922 34700170

[B16] D'Anna C. Cigna D. Di Sano C. Di Vincenzo S. Dino P. Ferraro M.. (2017). Exposure to cigarette smoke extract and lipopolysaccharide modifies cytoskeleton organization in bronchial epithelial cells. Exp. Lung Res. 43, 347–358. 10.1080/01902148.2017.1377784 29199880

[B17] Davuluri G. Allawy A. Thapaliya S. Rennison J. H. Singh D. Kumar A.. (2016). Hyperammonaemia-induced skeletal muscle mitochondrial dysfunction results in cataplerosis and oxidative stress. J. Physiol. 594, 7341–7360. 10.1113/JP272796 27558544 PMC5157075

[B18] Dingjan I. Paardekooper L. M. Verboogen D. R. J. von Mollard G. F. Ter Beest M. van den Bogaart G.. (2017). Vamp8-mediated Nox2 recruitment to endosomes is necessary for antigen release. Eur. J. Cell Biol. 96, 705–714. 10.1016/j.ejcb.2017.06.007 28688576 PMC5641923

[B19] Dong X. Liu Q. Kan D. Zhao W. Guo H. Lv L.. (2020). Effects of ammonia-N exposure on the growth, metabolizing enzymes, and metabolome of *Macrobrachium rosenbergii*. Ecotoxicol. Environ. Saf. 189, 110046. 10.1016/j.ecoenv.2019.110046 31835043

[B20] Drews L. Zimmermann M. Westhoff P. Brilhaus D. Poss R. E. Bergmann L.. (2020). Ammonia inhibits energy metabolism in astrocytes in a rapid and glutamate dehydrogenase 2-dependent manner. Dis. Model. Mech. 13. 10.1242/dmm.047134 32917661 PMC7657470

[B21] Duan Y. Liu Q. Wang Y. Zhang J. Xiong D. (2018). Impairment of the intestine barrier function in *Litopenaeus vannamei* exposed to ammonia and nitrite stress. Fish Shellfish Immunol. 78, 279–288. 10.1016/j.fsi.2018.04.050 29709590

[B22] Duan Y. Xiong D. Wang Y. Li H. Dong H. Zhang J.. (2021). Toxic effects of ammonia and thermal stress on the intestinal microbiota and transcriptomic and metabolomic responses of *Litopenaeus vannamei*. Sci. Total Environ. 754, 141867. 10.1016/j.scitotenv.2020.141867 32898779

[B23] Fang S. Chen X. Ye X. Zhou L. Xue S. Gan Q.. (2020). Effects of gut microbiome and short-chain fatty acids (scfas) on finishing weight of meat rabbits. Front. Microbiol. 11, 1835. 10.3389/fmicb.2020.01835 32849435 PMC7431612

[B24] Forsythe S. J. Parker D. S. (1985). Nitrogen metabolism by the microbial flora of the rabbit caecum. J. Appl. Bacteriol. 58, 363–369. 10.1111/j.1365-2672.1985.tb01475.x 3997689

[B25] Gibson S. A. Macfarlane G. T. (1988). Studies on the proteolytic activity of bacteroides fragilis. J. Gen. Microbiol. 134, 19–27. 10.1099/00221287-134-1-19 3053970

[B26] Gomez D. E. Arroyo L. G. Lillie B. Weese J. S. (2021). Nasal bacterial microbiota during an outbreak of equine herpesvirus 1 at a farm in Southern Ontario. Can. J. Vet. Res. 85, 3–11. 33390647 PMC7747660

[B27] Guttenberg M. A. Vose A. T. Tighe R. M. (2021). Role of innate immune system in environmental lung diseases. Curr. Allergy Asthma Rep. 21, 34. 10.1007/s11882-021-01011-0 33970346 PMC8311569

[B28] Hakvoort T. B. He Y. Kulik W. Vermeulen J. L. Duijst S. Ruijter J. M.. (2017). Pivotal role of glutamine synthetase in ammonia detoxification. Hepatology 65, 281–293. 10.1002/hep.28852 27641632

[B29] Han H. Zhou Y. Liu Q. Wang G. Feng J. Zhang M.. (2021). Effects of ammonia on gut microbiota and growth performance of broiler chickens. Animals 11:1716. 10.3390/ani11061716 34201291 PMC8228959

[B30] Han Q. Liu H. Zhang R. Yang X. Bao J. Xing H.. (2021). Selenomethionine protects against ammonia-induced apoptosis through inhibition of endoplasmic reticulum stress in pig kidneys. Ecotoxicol. Environ. Saf. 223, 112596. 10.1016/j.ecoenv.2021.112596 34352572

[B31] Heintz-Buschart A. Pandey U. Wicke T. Sixel-Doring F. Janzen A. Sittig-Wiegand E.. (2018). The nasal and gut microbiome in Parkinson's disease and idiopathic rapid eye movement sleep behavior disorder. Mov. Disord. 33, 88–98. 10.1002/mds.27105 28843021 PMC5811909

[B32] Hoche F. Klapperstuck T. Wohlrab J. (2004). Effects of L-ornithine on metabolic processes of the urea cycle in human keratinocytes. Skin Pharmacol. Physiol. 17, 283–288. 10.1159/000081113 15528958

[B33] Hongxing G. Xiafei L. Jialing L. Zhenquan C. Luoyu G. Lei L.. (2021). Effects of acute ammonia exposure on antioxidant and detoxification metabolism in clam *Cyclina sinensis*. Ecotoxicol. Environ. Saf. 211, 111895. 10.1016/j.ecoenv.2021.111895 33476851

[B34] Jandhyala S. M. Talukdar R. Subramanyam C. Vuyyuru H. Sasikala M. Nageshwar Reddy D.. (2015). Role of the normal gut microbiota. World J. Gastroenterol. 21, 8787–8803. 10.3748/wjg.v21.i29.8787 26269668 PMC4528021

[B35] Jin D. X. Zou H. W. Liu S. Q. Wang L. Z. Xue B. Wu D.. (2018). The underlying microbial mechanism of epizootic rabbit enteropathy triggered by a low fiber diet. Sci. Rep. 8, 12489. 10.1038/s41598-018-30178-2 30131509 PMC6104036

[B36] Jin L. Li D. Alesi G. N. Fan J. Kang H. B. Lu Z.. (2015). Glutamate dehydrogenase 1 signals through antioxidant glutathione peroxidase 1 to regulate redox homeostasis and tumor growth. Cancer Cell 27, 257–270. 10.1016/j.ccell.2014.12.006 25670081 PMC4325424

[B37] Katunuma N. Okada M. Nishii Y. (1966). Regulation of the urea cycle and tca cycle by ammonia. Adv. Enzyme Regul. 4, 317–336. 10.1016/0065-2571(66)90025-2 4229888

[B38] Ke S. Fang S. He M. Huang X. Yang H. Yang B.. (2019). Age-based dynamic changes of phylogenetic composition and interaction networks of health pig gut microbiome feeding in a uniformed condition. BMC Vet. Res. 15, 172. 10.1186/s12917-019-1918-5 31126262 PMC6534858

[B39] Keulers L. Dehghani A. Knippels L. Garssen J. Papadopoulos N. Folkerts G.. (2022). Probiotics, prebiotics, and synbiotics to prevent or combat air pollution consequences: the gut-lung axis. Environ. Pollut. 302, 119066. 10.1016/j.envpol.2022.119066 35240267

[B40] Kilic I. Simsek E. Yaslioglu E. Heber A. Uguz S. (2021). Air quality measurements in four sheep barns part II: pollutant gas emissions. Environ. Sci. Pollut. Res. Int. 28, 19064–19078. 10.1007/s11356-020-12184-y 33394430

[B41] Kim H. K. Lee G. H. Bhattarai K. R. Lee M. S. Back S. H. Kim H. R.. (2021). Tmbim6 (transmembrane bax inhibitor motif containing 6) enhances autophagy through regulation of lysosomal calcium. Autophagy 17, 761–778. 10.1080/15548627.2020.1732161 32167007 PMC8032251

[B42] Kim S. Goel R. Kumar A. Qi Y. Lobaton G. Hosaka K.. (2018). Imbalance of gut microbiome and intestinal epithelial barrier dysfunction in patients with high blood pressure. Clin. Sci. 132, 701–718. 10.1042/CS20180087 29507058 PMC5955695

[B43] Kim W. G. Kang G. D. Kim H. I. Han M. J. Kim D. H. (2019). *Bifidobacterium longum* Im55 and *Lactobacillus plantarum* Im76 alleviate allergic rhinitis in mice by restoring Th2/Treg imbalance and gut microbiota disturbance. Benef. Microbes 10, 55–67. 10.3920/BM2017.0146 30465441

[B44] Lee J. J. Kim S. H. Lee M. J. Kim B. K. Song W. J. Park H. W.. (2019). Different upper airway microbiome and their functional genes associated with asthma in young adults and elderly individuals. Allergy 74, 709–719. 10.1111/all.13608 30242844

[B45] Li L. H. Qi H. X. (2019). Effect of acute ammonia exposure on the glutathione redox system in Ffrc strain common carp (*Cyprinus carpio*, L.). Environ. Sci. Pollut. Res. Int. 26, 27023–27031. 10.1007/s11356-019-05895-4 31313232

[B46] Li Y. Pan L. Zeng X. Zhang R. Li X. Li J.. (2021a). Ammonia exposure causes the imbalance of the gut-brain axis by altering gene networks associated with oxidative metabolism, inflammation and apoptosis. Ecotoxicol. Environ. Saf. 224, 112668. 10.1016/j.ecoenv.2021.112668 34450428

[B47] Li Y. Yan P. Lei Q. Li B. Sun Y. Li S.. (2019). Metabolic adaptability shifts of cell membrane fatty acids of *Komagataeibacter hansenii* Hdm1-3 improve acid stress resistance and survival in acidic environments. J. Ind. Microbiol. Biotechnol. 46, 1491–1503. 10.1007/s10295-019-02225-y 31512094

[B48] Li Y. Zhang R. Li X. Li J. Ji W. Zeng X.. (2021b). Exposure to the environmental pollutant ammonia causes changes in gut microbiota and inflammatory markers in fattening pigs. Ecotoxicol. Environ. Saf. 208, 111564. 10.1016/j.ecoenv.2020.111564 33396094

[B49] Liu Q. X. Zhou Y. Li X. M. Ma D. D. Xing S. Feng J. H.. (2020). Ammonia induce lung tissue injury in broilers by activating Nlrp3 inflammasome via Escherichia/Shigella. Poult. Sci. 99, 3402–3410. 10.1016/j.psj.2020.03.019 32616234 PMC7597683

[B50] Lopez-Serrano S. Galofre-Mila N. Costa-Hurtado M. Perez-de-Rozas A. M. Aragon V. (2020). Heterogeneity of moraxella isolates found in the nasal cavities of piglets. BMC Vet. Res. 16, 28. 10.1186/s12917-020-2250-9 32000773 PMC6993494

[B51] Louis P. Flint H. J. (2017). Formation of propionate and butyrate by the human colonic microbiota. Environ. Microbiol. 19, 29–41. 10.1111/1462-2920.13589 27928878

[B52] Lv X. Chai J. Diao Q. Huang W. Zhuang Y. Zhang N.. (2019). The signature microbiota drive rumen function shifts in goat kids introduced to solid diet regimes. Microorganisms 7:516. 10.3390/microorganisms7110516 31683646 PMC6921049

[B53] Ma S. Zhang F. Zhou F. Li H. Ge W. Gan R.. (2021). Metagenomic analysis reveals oropharyngeal microbiota alterations in patients with Covid-19. Signal Transd. Target. Therapy 6, 191. 10.1038/s41392-021-00614-3 33986253 PMC8116522

[B54] Martins I. Galluzzi L. Kroemer G. (2011). Hormesis, cell death and aging. Aging 3, 821–828. 10.18632/aging.100380 21931183 PMC3227447

[B55] McCauley K. Durack J. Valladares R. Fadrosh D. W. Lin D. L. Calatroni A.. (2019). Distinct nasal airway bacterial microbiotas differentially relate to exacerbation in pediatric patients with asthma. J. Allergy Clin. Immunol. 144, 1187–1197. 10.1016/j.jaci.2019.05.035 31201890 PMC6842413

[B56] Morawska-Kochman M. Jermakow K. Nelke K. Zub K. Pawlak W. Dudek K.. (2019). The Ph value as a factor modifying bacterial colonization of sinonasal mucosa in healthy persons. Ann. Otol. Rhinol. Laryngol. 128, 819–828. 10.1177/0003489419843143 31014081

[B57] Mousavi S. E. Delgado-Saborit J. M. Adivi A. Pauwels S. Godderis L. (2022). Air pollution and endocrine disruptors induce human microbiome imbalances: a systematic review of recent evidence and possible biological mechanisms. Sci. Total Environ. 816, 151654. 10.1016/j.scitotenv.2021.151654 34785217

[B58] Park J. W. Jeong J. S. Lee S. I. Kim I. H. (2016). Effect of dietary supplementation with a probiotic (*Enterococcus faecium*) on production performance, excreta microflora, ammonia emission, and nutrient utilization in isa brown laying hens. Poult. Sci. 95, 2829–2835. 10.3382/ps/pew241 27422665

[B59] Philipp T. M. Will A. Richter H. Winterhalter P. R. Pohnert G. Steinbrenner H.. (2021). A coupled enzyme assay for detection of selenium-binding protein 1 (Selenbp1) methanethiol oxidase (Mto) activity in mature enterocytes. Redox Biol. 43, 101972. 10.1016/j.redox.2021.101972 33901808 PMC8099554

[B60] Pokharel B. B. Dos Santos V. M. Wood D. Van Heyst B. Harlander-Matauschek A. (2017). Laying hens behave differently in artificially and naturally sourced ammoniated environments. Poult. Sci. 96, 4151–4157. 10.3382/ps/pex273 29053839

[B61] Premachandra H. K. A. Elvitigala D. A. S. Whang I. Kim E. De Zoysa M. Lim B. S.. (2013). Expression profile of cystatin B ortholog from manila clam (*Ruditapes philippinarum*) in host pathology with respect to its structural and functional properties. Fish Shellfish Immunol. 34, 1505–1513. 10.1016/j.fsi.2013.03.349 23528873

[B62] Qin T. Zhang F. Zhou H. Ren H. Du Y. Liang S.. (2019). High-level Pm2.5/Pm10 exposure is associated with alterations in the human pharyngeal microbiota composition. Front. Microbiol. 10, 54. 10.3389/fmicb.2019.00054 30804895 PMC6379047

[B63] Qin W. Shen L. Wang Q. Gao Y. She M. Li X.. (2022). Chronic exposure to ammonia induces oxidative stress and enhanced glycolysis in lung of piglets. Environ. Toxicol. 37, 179–191. 10.1002/tox.23382 34806272

[B64] Rodriguez-Palacios A. Harding A. Menghini P. Himmelman C. Retuerto M. Nickerson K. P.. (2018). The artificial sweetener splenda promotes gut proteobacteria, dysbiosis, and myeloperoxidase reactivity in crohn's disease-like ileitis. Inflamm. Bowel Dis. 24, 1005–1020. 10.1093/ibd/izy060 29554272 PMC5950546

[B65] Shin T. H. Seo C. Lee D. Y. Ji M. Manavalan B. Basith S.. (2019). Silica-coated magnetic nanoparticles induce glucose metabolic dysfunction *in vitro* via the generation of reactive oxygen species. Arch. Toxicol. 93, 1201–1212. 10.1007/s00204-019-02402-z 30737549

[B66] Sies H. Jones D. P. (2020). Reactive oxygen species (ros) as pleiotropic physiological signalling agents. Nat. Rev. Mol. Cell Biol. 21, 363–383. 10.1038/s41580-020-0230-3 32231263

[B67] Stancik L. M. Stancik D. M. Schmidt B. Barnhart D. M. Yoncheva Y. N. Slonczewski J. L.. (2002). Ph-dependent expression of periplasmic proteins and amino acid catabolism in *Escherichia coli*. J. Bacteriol. 184, 4246–4258. 10.1128/JB.184.15.4246-4258.2002 12107143 PMC135203

[B68] Su H. Na N. Zhang X. Zhao Y. (2017). The biological function and significance of Cd74 in immune diseases. Inflamm. Res. 66, 209–216. 10.1007/s00011-016-0995-1 27752708

[B69] Sun H. Wang W. Li J. Yang Z. (2014). Growth, oxidative stress responses, and gene transcription of juvenile bighead carp *(Hypophthalmichthys nobilis*) under chronic-term exposure of ammonia. Environ. Toxicol. Chem. 33, 1726–1731. 10.1002/etc.2613 24839064

[B70] Takahashi K. Koga K. Linge H. M. Zhang Y. Lin X. Metz C. N.. (2009). Macrophage Cd74 contributes to mif-induced pulmonary inflammation. Respir. Res. 10, 33. 10.1186/1465-9921-10-33 19413900 PMC2681459

[B71] Tang S. Xie J. Wu W. Yi B. Liu L. Zhang H.. (2020). High ammonia exposure regulates lipid metabolism in the pig skeletal muscle via mtor pathway. Sci. Total Environ. 740, 139917. 10.1016/j.scitotenv.2020.139917 32563870

[B72] Tang S. Zhong R. Yin C. Su D. Xie J. Chen L.. (2021b). Exposure to high aerial ammonia causes hindgut dysbiotic microbiota and alterations of microbiota-derived metabolites in growing pigs. Front. Nutr. 8, 689818. 10.3389/fnut.2021.689818 34179063 PMC8231926

[B73] Tao Z. Xu W. Zhu C. Zhang S. Shi Z. Song W.. (2019). Effects of ammonia on intestinal microflora and productive performance of laying ducks. Poult. Sci. 98, 1947–1959. 10.3382/ps/pey578 30649519

[B74] Thiele Orberg E. Fan H. Tam A. J. Dejea C. M. Destefano Shields C. E. Wu S.. (2017). The myeloid immune signature of enterotoxigenic bacteroides fragilis-induced murine colon tumorigenesis. Mucosal Immunol. 10, 421–433. 10.1038/mi.2016.53 27301879 PMC5159334

[B75] Ticinesi A. Lauretani F. Tana C. Nouvenne A. Ridolo E. Meschi T.. (2019). Exercise and immune system as modulators of intestinal microbiome: implications for the gut-muscle axis hypothesis. Exerc. Immunol. Rev. 25, 84–95. 30753131

[B76] Urbain B. Gustin P. Charlier G. Coignoul F. Lambotte J. L. Grignon G.. (1996). A morphometric and functional study of the toxicity of atmospheric ammonia in the extrathoracic airways in pigs. Vet. Res. Commun. 20, 381–399. 10.1007/BF00366545 8865581

[B77] Voss C. M. Arildsen L. Nissen J. D. Waagepetersen H. S. Schousboe A. Maechler P.. (2021). Glutamate dehydrogenase is important for ammonia fixation and amino acid homeostasis in brain during hyperammonemia. Front. Neurosci. 15, 646291. 10.3389/fnins.2021.646291 34220417 PMC8244593

[B78] Wang G. Liu Q. Zhou Y. Feng J. Zhang M. (2022). Effects of different ammonia concentrations on pulmonary microbial flora, lung tissue mucosal morphology, inflammatory cytokines, and neurotransmitters of broilers. Animals 12:261. 10.3390/ani12030261 35158583 PMC8833639

[B79] Wang H. Zeng X. Zhang X. Liu H. Xing H. (2021). Ammonia exposure induces oxidative stress and inflammation by destroying the microtubule structures and the balance of solute carriers in the trachea of pigs. Ecotoxicol. Environ. Saf. 212, 111974. 10.1016/j.ecoenv.2021.111974 33508713

[B80] Wang L. Sun M. Lin X. Lei Y. Yin Z. Zhou W.. (2021). Down-regulation of Hbxip inhibits non-small cell lung cancer growth and enhances the anti-tumor immunity of mice by reducing Nrp-1. Ann. Clin. Lab. Sci. 51, 487–493. 34452886

[B81] Wang S. Li X. Zhang M. Jiang H. Wang R. Qian Y.. (2021). Ammonia stress disrupts intestinal microbial community and amino acid metabolism of juvenile yellow catfish (*Pelteobagrus fulvidraco*). Ecotoxicol. Environ. Saf. 227, 112932. 10.1016/j.ecoenv.2021.112932 34700169

[B82] Wang T. He Q. Yao W. Shao Y. Li J. Huang F.. (2019). The variation of nasal microbiota caused by low levels of gaseous ammonia exposure in growing pigs. Front. Microbiol. 10, 1083. 10.3389/fmicb.2019.01083 31156592 PMC6532555

[B83] Wang X. Wang M. Chen S. Wei B. Gao Y. Huang L.. (2020). Ammonia exposure causes lung injuries and disturbs pulmonary circadian clock gene network in a pig study. Ecotoxicol. Environ. Saf. 205, 111050. 10.1016/j.ecoenv.2020.111050 32827960

[B84] Wu D. Zhang M. Xu J. Song E. Lv Y. Tang S.. (2016). *In vitro* evaluation of aspirin-induced hspb1 against heat stress damage in chicken myocardial cells. Cell Stress Chaper. 21, 405–413. 10.1007/s12192-016-0666-8 26910344 PMC4837179

[B85] Wu S. E. Hashimoto-Hill S. Woo V. Eshleman E. M. Whitt J. Engleman L.. (2020). Microbiota-derived metabolite promotes Hdac3 activity in the gut. Nature 586, 108–112. 10.1038/s41586-020-2604-2 32731255 PMC7529926

[B86] Wu X. Luo Q. Liu Z. (2020). Ubiquitination and deubiquitination of mcl1 in cancer: deciphering chemoresistance mechanisms and providing potential therapeutic options. Cell Death Dis. 11, 556. 10.1038/s41419-020-02760-y 32699213 PMC7376237

[B87] Xia Y. Liu N. Xie X. Bi G. Ba H. Li L.. (2019). The Macrophage-specific V-atpase subunit Atp6v0d2 restricts inflammasome activation and bacterial infection by facilitating autophagosome-lysosome fusion. Autophagy 15, 960–975. 10.1080/15548627.2019.1569916 30681394 PMC6526827

[B88] Yeh W. L. Hsu Y. J. Ho C. S. Ho H. H. Kuo Y. W. Tsai S. Y.. (2022). *Lactobacillus plantarum* Pl-02 supplementation combined with resistance training improved muscle mass, force, and exercise performance in mice. Fron.t Nutr. 9, 896503. 10.3389/fnut.2022.896503 35571912 PMC9094439

[B89] Yin H. Zhong Y. Wang H. Hu J. Xia S. Xiao Y.. (2022). Short-term exposure to high relative humidity increases blood urea and influences colonic urea-nitrogen metabolism by altering the gut microbiota. J. Adv. Res. 35, 153–168. 10.1016/j.jare.2021.03.004 35003799 PMC8721250

[B90] Zhang X. Yang F. Zhang X. Xu Y. Liao T. Song S.. (2008). Induction of hepatic enzymes and oxidative stress in Chinese rare minnow (*Gobiocypris rarus*) exposed to waterborne hexabromocyclododecane (Hbcdd). Aquat. Toxicol. 86, 4–11. 10.1016/j.aquatox.2007.07.002 18022707

[B91] Zheng F. Goncalves F. M. Abiko Y. Li H. Kumagai Y. Aschner M.. (2020). Redox toxicology of environmental chemicals causing oxidative stress. Redox Biol. 34, 101475. 10.1016/j.redox.2020.101475 32336668 PMC7327986

[B92] Zhou Y. Zhang M. Zhao X. Feng J. (2021). Ammonia exposure induced intestinal inflammation injury mediated by intestinal microbiota in broiler chickens via Tlr4/Tnf-alpha signaling pathway. Ecotoxicol. Environ. Saf. 226, 112832. 10.1016/j.ecoenv.2021.112832 34583273

[B93] Zhu L. Xu L. Z. Zhao S. Shen Z. F. Shen H. Zhan L. B.. (2020). Protective effect of baicalin on the regulation of Treg/Th17 balance, gut microbiota and short-chain fatty acids in rats with ulcerative colitis. Appl. Microbiol. Biotechnol. 104, 5449–5460. 10.1007/s00253-020-10527-w 32322944

