# Supplementary figures and images for: House ammonia exposure causes alterations in microbiota, transcriptome, and metabolome of rabbits
Source: Front Microbiol. 2023 May 12;14:1125195. doi: 10.3389/fmicb.2023.1125195 (PMC10213413; doi:10.3389/fmicb.2023.1125195)

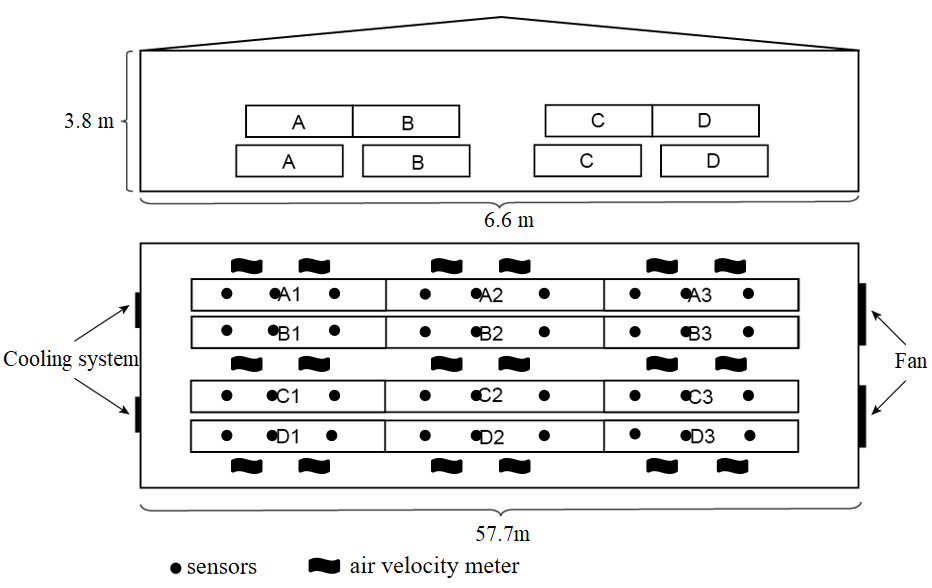

Supplement: Supplementary Figure S1 — Diagram of confined barn structural parameters and air quality measurement instrument distribution. A, B, C, and D represent four sets of double-deck cages, which were further divided evenly into three parts (1, 2, and 3). [file Data_Sheet_1.ZIP › Supplementary Material/Fig S1.tiff]

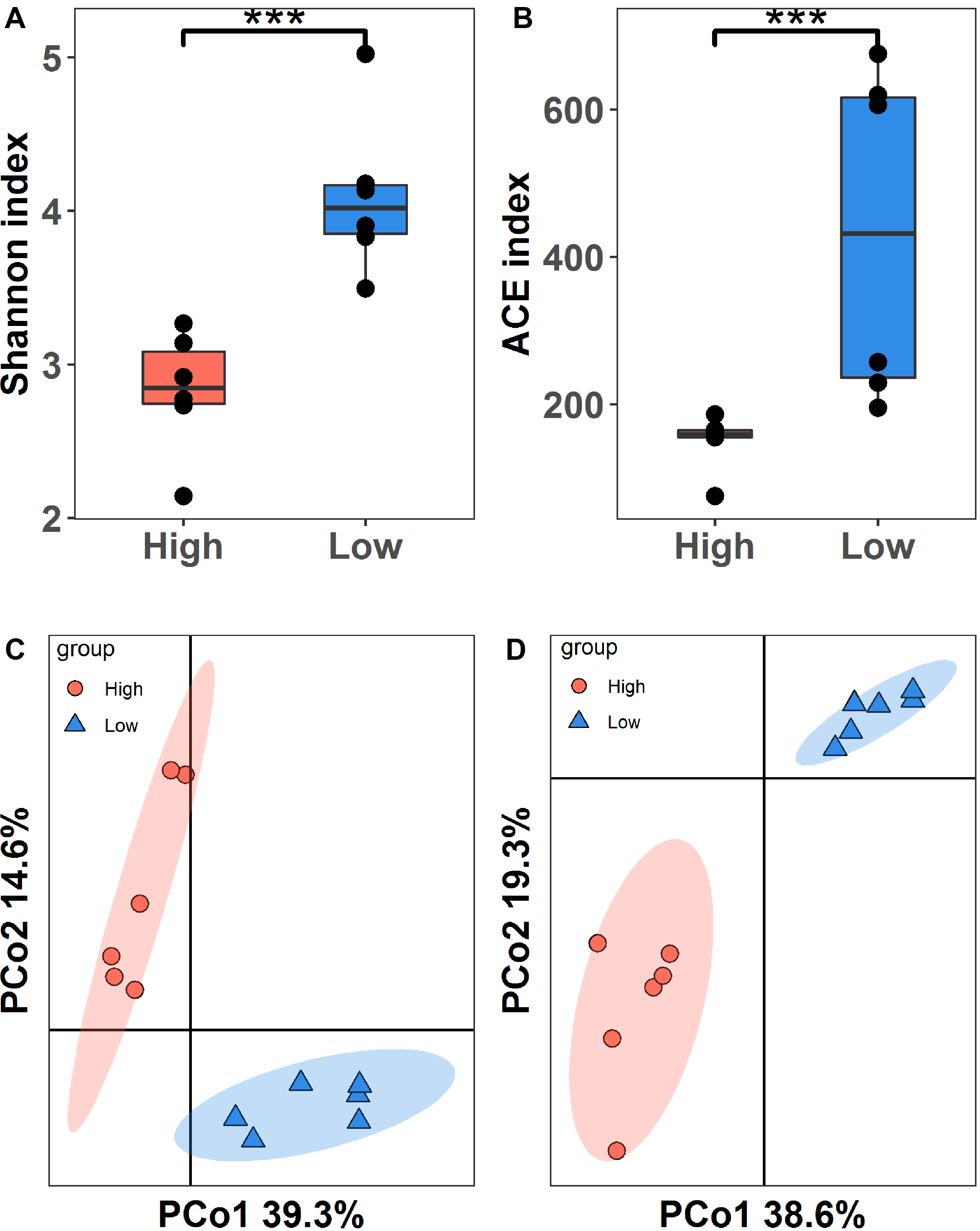

Supplement: Supplementary Figure S1 — Diagram of confined barn structural parameters and air quality measurement instrument distribution. A, B, C, and D represent four sets of double-deck cages, which were further divided evenly into three parts (1, 2, and 3). [file Data_Sheet_1.ZIP › Supplementary Material/Fig S2.tiff]

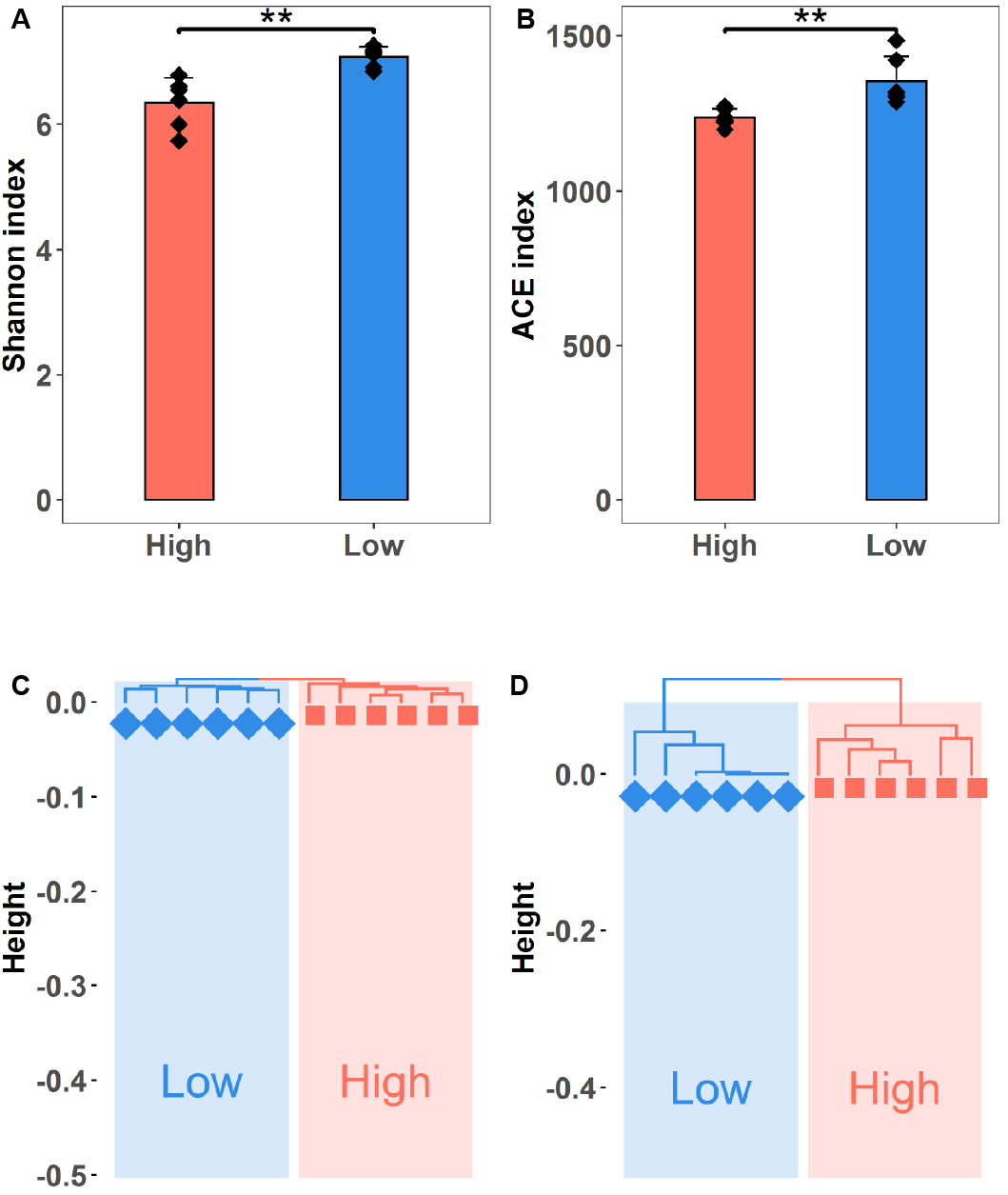

Supplement: Supplementary Figure S1 — Diagram of confined barn structural parameters and air quality measurement instrument distribution. A, B, C, and D represent four sets of double-deck cages, which were further divided evenly into three parts (1, 2, and 3). [file Data_Sheet_1.ZIP › Supplementary Material/Fig S3.tiff]

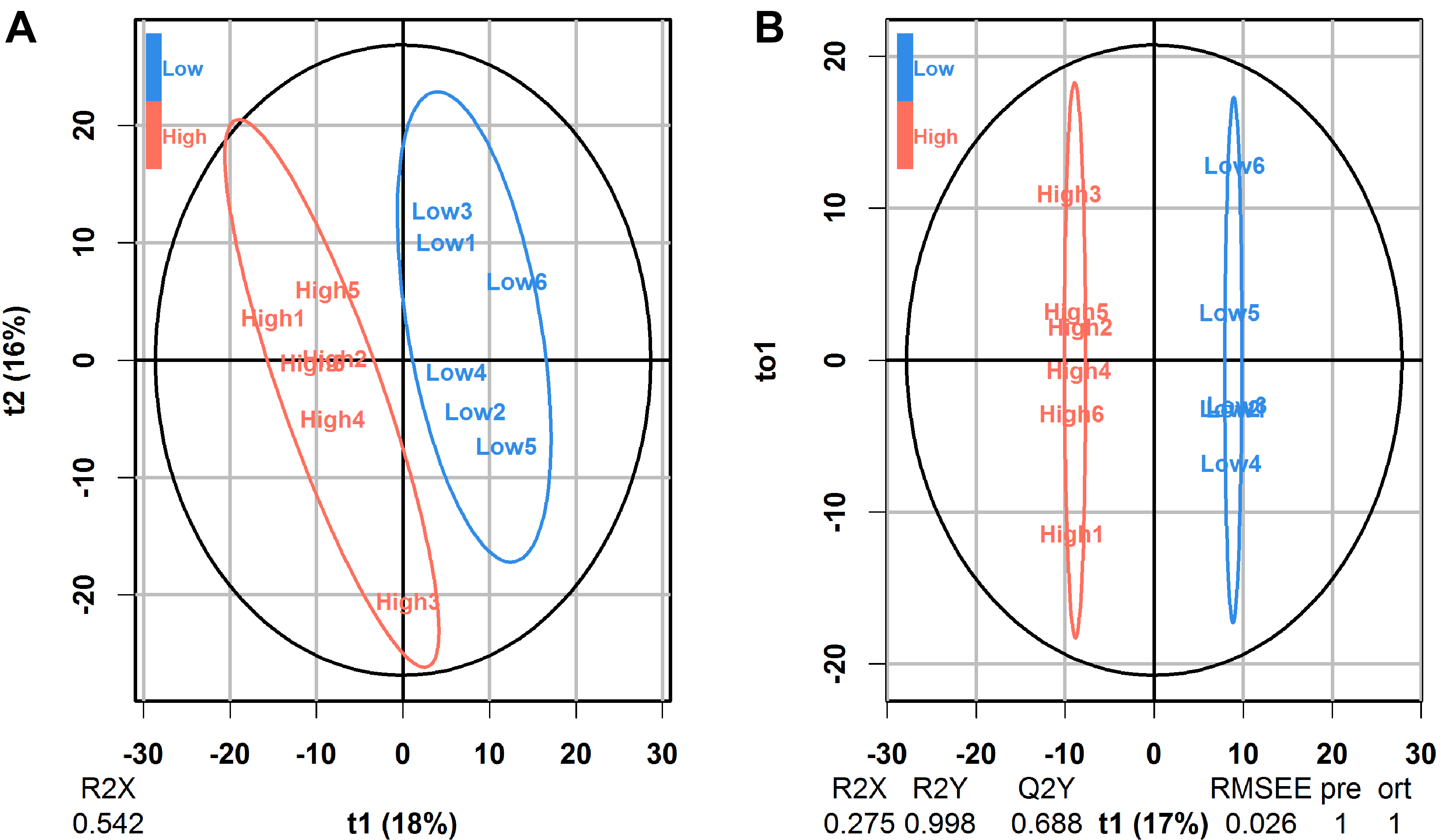

Supplement: Supplementary Figure S1 — Diagram of confined barn structural parameters and air quality measurement instrument distribution. A, B, C, and D represent four sets of double-deck cages, which were further divided evenly into three parts (1, 2, and 3). [file Data_Sheet_1.ZIP › Supplementary Material/Fig S4.tiff]

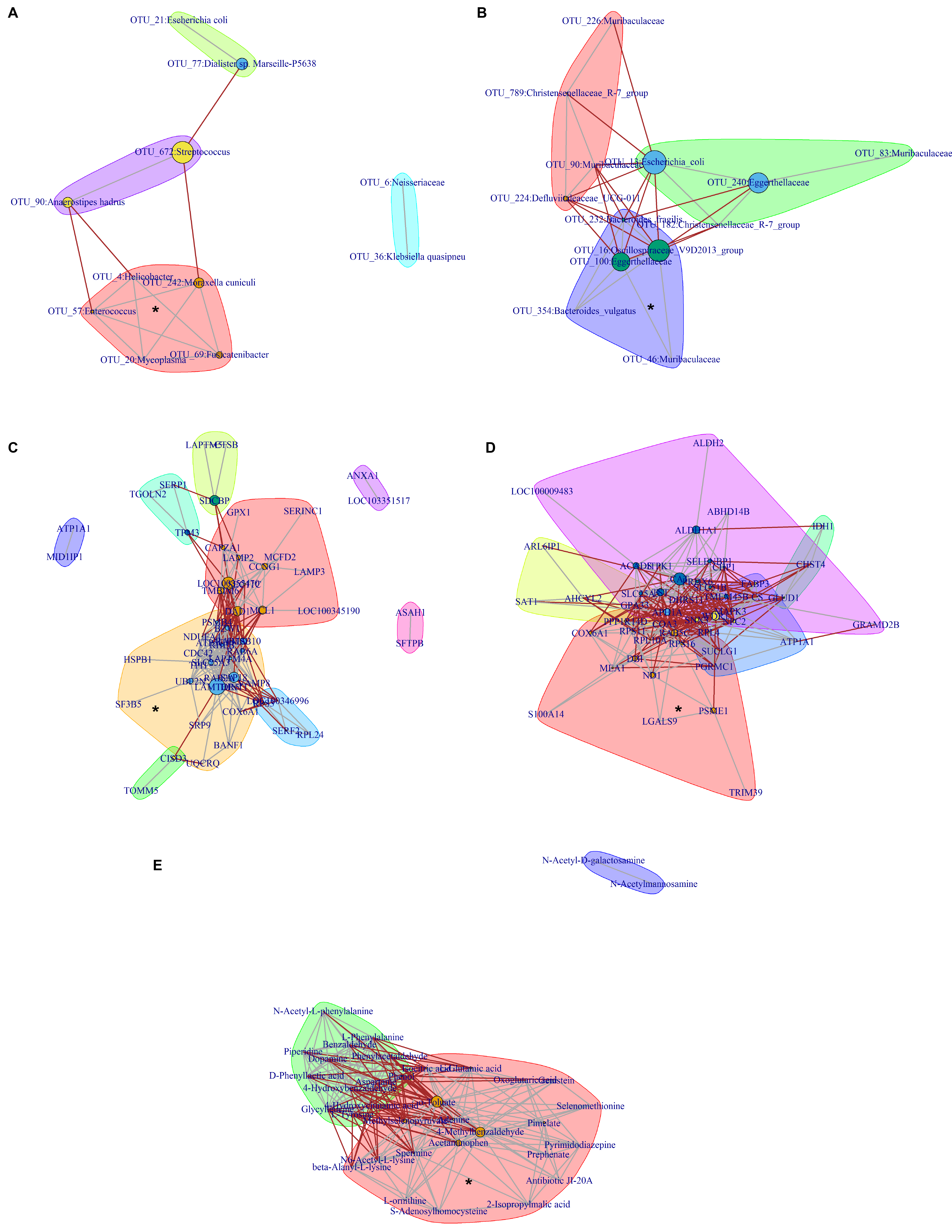

Supplement: Supplementary Figure S1 — Diagram of confined barn structural parameters and air quality measurement instrument distribution. A, B, C, and D represent four sets of double-deck cages, which were further divided evenly into three parts (1, 2, and 3). [file Data_Sheet_1.ZIP › Supplementary Material/Fig S5.tiff]
